# Screening of a Plant Extract Library from the Greek Flora for Biological Activities Related to Anti-Aging Applications

**DOI:** 10.3390/antiox14070824

**Published:** 2025-07-04

**Authors:** Harris Pratsinis, Despoina D. Gianniou, Gabriela Belén Lemus Ringele, Adamantia Agalou, Asimina Fotopoulou, Xanthippi P. Louka, Christos Nastos, Eleftherios Kalpoutzakis, Aikaterini Argyropoulou, Dimitris Michailidis, Antonia Theodoridi, Ioanna Eleftheriadou, Adamantia Papadopoulou, Sentiljana Gumeni, Stavros Beteinakis, Konstantina Karamanou, Eleni Mavrogonatou, Georgios Stavropoulos, Dimitris Beis, Maria Halabalaki, Ioannis P. Trougakos, Dimitris Kletsas

**Affiliations:** 1 Laboratory of Cell Proliferation & Ageing, Institute of Biosciences & Applications, NCSR “Demokritos”, 15341 Athens, Greece; hprats@bio.demokritos.gr (H.P.); asiminaf@bio.demokritos.gr (A.F.); apapad@bio.demokritos.gr (A.P.); karamanou@hbio.gr (K.K.); elmavro@bio.demokritos.gr (E.M.); 2 Department of Cell Biology and Biophysics, Faculty of Biology, National and Kapodistrian University of Athens, 15784 Athens, Greece; gndespoina@biol.uoa.gr (D.D.G.); x.louka@biol.uoa.gr (X.P.L.); sgumeni@biol.uoa.gr (S.G.); itrougakos@biol.uoa.gr (I.P.T.); 3 Division of Pharmacognosy and Natural Products Chemistry, Department of Pharmacy, National and Kapodistrian University of Athens, 15771 Athens, Greece; glemus@pharm.uoa.gr (G.B.L.R.); elkalp@pharm.uoa.gr (E.K.); kargyropoulou@pharmagnose.com (A.A.); sbeteinakis@pharm.uoa.gr (S.B.); mariahal@pharm.uoa.gr (M.H.); 4 Center for Clinical, Experimental Surgery & Translational Research, Biomedical Research Foundation, Academy of Athens, 11527 Athens, Greece; a.agalou@gmail.com (A.A.); tonia.theod@gmail.com (A.T.); dbeis@uoi.gr (D.B.); 5 PharmaGnose S.A., 57th km Athens-Lamia Road, 32011 Oinofyta Viotia, Greece; nastos@pharmagnose.com (C.N.); michailidis@pharmagnose.com (D.M.); 6 Laboratory of Biological Chemistry, School of Health Sciences, Faculty of Medicine, University of Ioannina, 45110 Ioannina, Greece; bl02413@uoi.gr; 7 Korres S.A.—Natural Products, 57th km Athens-Lamia Road, 32011 Oinofyta Viotia, Greece; giorgos.stavropoulos@korres.com

**Keywords:** skin aging, plant biodiversity, accelerated solvent extraction, supercritical fluid extraction, reactive oxygen species, collagenase, elastase, tyrosinase, proteaseome, zebrafish

## Abstract

Characteristic manifestations of skin aging, due to either intrinsic or extrinsic factors, such as ultraviolet (UV) radiation and oxidative stress, include cell senescence, alterations in collagen and elastin networks, and melanogenesis disorders. Natural products are considered a rich source of anti-aging molecules. Accordingly, the screening of a plant extract library from the Greek flora for a panel of biological activities related to skin aging is presented herein. In particular, 52 plant materials extracted using Accelerated Solvent Extraction (ASE) and Supercritical Fluid Extraction (SFE) were assessed for their effects on (1) human skin cell viability, (2) antioxidant activity—using both cell-free and cell-based methods—(3) photoprotective capacity, and (4) interference with collagenase, elastase, and tyrosinase, as well as with proteasomal and lysosomal activities of human skin cells. *In vivo* phenotypic screens on *Danio rerio* (zebrafish) embryos were also used for assessing melanogenesis. Many active extracts were identified, some of them for the first time, and others in agreement with previous reports. In general, ASE extracts exhibited higher activities than SFE ones. Seven extracts showed multiple activities, being highly effective in at least four different assays. These data support the potential use of these extracts against skin aging in medicinal and cosmetic applications.

## 1. Introduction

Skin is the largest organ of the human body and due to its outermost anatomical location assumes—among many other functions—the role of the organism’s boundary and first defense against environmental insults [1]. Skin also usually provides the best representation of organismal aging. Skin aging is affected by a combination of endogenous or intrinsic (genetics, cellular metabolism, hormones, and metabolic processes) and exogenous or extrinsic (light exposure, pollution, ionizing radiation, chemicals, and toxins) parameters [2]. Among the latter, one of the most important factors is ultraviolet (UV) radiation, leading to a characteristic type of skin aging called photoaging [3]. Central features of the aged skin are the progressive loss of cells, as well as alterations in the composition and organization of extracellular matrix (ECM), especially regarding the collagen and elastin networks, leading to the formation of wrinkles [4]. Moreover, discoloration and abnormalities in skin pigmentation (such as senile lentigo and melasma) are quite often observed in the elderly skin [5,6]. Many mechanisms have been reported to underly skin aging manifestations, extremely important ones being oxidative stress and the loss of balance between anabolic and catabolic processes [4,7,8]. Moreover, accumulation of senescent cells has been reported to contribute to skin aging, mainly due to the senescence-associated secretory phenotype (SASP) [9,10] but also through other mechanisms, such as DNA damage-induced melanogenesis [11]. Hence, it is not surprising that a plethora of strategies have been employed against skin aging [2], the most popular among them being the use of medicinal and cosmetic products either to prevent skin damage (e.g., protection from sunlight) or to revert age-related changes (e.g., to enhance collagen production and/or inhibit collagenolytic processes).

The quest of humankind for active molecules in order to enrich our arsenal of pharmaceutics and cosmetics is based both on synthetic approaches and on natural products. The latter—either of plant, marine, or microbial origin—have been the most productive source of leads for the development of drugs, since they exhibit a broad range of structural diversity, being structurally evolved to play specific biological roles [12]. In particular, plants have been used in cosmetic formulations since ancient times, while nowadays, plant-derived ingredients are in increasing demand from the cosmetic and pharmaceutical industries [13]. Greece is known for its unique biodiversity, consisting of approximately 6000 species of higher plants. Of these, ~15% of the plant taxa are endemic, making Greece an area of high conservation priority [14,15]. Due to its wealth, there is an increasing interest in the study of the Greek flora, since a great variety of bioactive compounds can be obtained. Following our previous study on plants endemic in Greece [16], the aim of the present work was to evaluate the chemical content and the biological properties of various plants of the Greek flora for their potential use in cosmetic applications against skin aging. In particular, 52 plant materials from different areas were selected, collected, and extracted using two techniques based on green chemistry principles, i.e., Supercritical Fluid Extraction (SFE-CO_2_) and Accelerated Solvent Extraction (ASE). The extracts were assessed for their activities regarding a broad spectrum of skin health-related parameters, i.e., viability of human skin cells, antioxidant activity—using both cell-free and cell-based methods—interference with collagenase, elastase, and tyrosinase activity, photoprotection, and proteasomal and lysosomal activities of human skin cells. Beyond these *in vitro* assays, the extracts were also tested by means of *Danio rerio* (zebrafish) *in vivo* phenotypic screens, which are increasingly used to identify novel bioactive compounds, especially in complex samples such as natural product extracts [17,18,19].

## 2. Materials and Methods

### 2.1. Plant Material

Samples from 41 genera and 49 different species of Greek flora belonging to 20 different families (Table 1) were collected from various regions across Greece. The botanical identification of the collected plants was performed by Dr. E. Kalpoutzakis from the Department of Pharmacy at the NKUA. Voucher specimens have been deposited at the Herbarium of the Division of Pharmacognosy and Natural Products Chemistry, Department of Pharmacy, NKUA.

### 2.2. Extraction

A. Accelerated Solvent Extraction (ASE): Extraction of samples was performed using an ASE 300 apparatus (Dionex, Sunnyvale, CA, USA) with a solvent mixture of distilled water and isopropanol (H_2_O:iPrOH, 1:1 *v*/*v*). A total of 20 g of dried plant material was placed into stainless-steel extraction cells, which were heated and extracted with the following conditions: preheating for 3 min at 40 °C with a pressure of 103 bar, heating cells for 5 min, 3 min static time, flush time 3 min, and purge time 120 s. Obtained extracts were evaporated to dryness using a rotary evaporator and were kept at −20 °C pending analysis. Samples derived from ASE were given the suffix “A” following their number (see Table 1).
B. Supercritical Fluid Extraction-CO_2_ (SFE-CO_2_): Extraction of samples was performed using an SFE (Separex, Champigneulles, France) system, using a mixture of carbon dioxide (CO_2_) and 5% of iPrOH as co-solvent. A total o f20 g of dried plant material was placed into a 100 mL stainless-steel extraction cell. CO_2_ was pumped (Piston pump P200 LGP50, Separex, France) through the system with a flow rate of 25 g/min, while iPrOH was pumped (Series III P300, Teledyne SSI, State College, PA, USA) with a flow rate of 1.58 mL/min. Oven and basket temperature were set at 40 °C and 35 °C, respectively, with a system pressure of 250 bar. Extracts were collected every 30 min for 2 h, and then were evaporated to dryness using a rotary evaporator. All extracts were kept at −20 °C pending analysis. Samples derived from SFE were given the suffix “B” following their number (see Table 1).

### 2.3. Chemical Analysis

#### Liquid Chromatography—High Resolution Mass Spectrometry (LC-HRMS/MS)

LC-MS analysis of obtained extracts both with SFE and ASE was performed using a Velos Pro Ion Trap-Orbitrap Elite Hybrid Mass Spectrometer system (Thermo Scientific, Bremen, Germany), with a heated electrospray ion source (HESI) hyphenated to a Waters Acquity UPLC (Waters Corporation, Milford, MA, USA) system. Separation was carried out on an Ascentis^®^ Express 90Å C18 column (15 cm × 2.1 mm, 2 um), using a mobile phase consisting of H_2_O with 0.1% *v*/*v* formic acid (A) and acetonitrile (B). The gradient method was set as follows: 5% B (0–1 min), 5–100% (1–15 min), 100% B (15–17 min), 100–5% B (17–17.5 min), 5% B (17.5–20 min). The total running time was 20 min. The column was kept at 40 °C during the analysis, and the flow rate was set to 0.3 mL/min. All samples were prepared at a final concentration of 300 μg/mL, and 10 μL was injected into the system. For the HESI source, capillary interface and source heater temperatures were set at 350 °C, while source electrospray voltage was 2.7 kV. S-Lens RF levels were tuned at 45% and 60% for negative and positive polarity, respectively. Data were obtained in negative and positive ionization mode, at a scan range of *m*/*z* 114–1000, with a resolution of 120,000. Nitrogen was used as sheath and auxiliary gas with flows set at 45 and 15 arbitrary units, respectively. A data-dependent acquisition (DDA) method was set up involving two scan events: a full MS scan followed by HRMS/MS acquisition. Full-scan spectra were acquired at a high resolution of 60,000 at full width half maximum (FWHM), whereas HRMS/MS acquisition were acquired at a mass resolution of 30,000 at FWHM. Higher-energy collisional dissociation (HCD) resulted in normalized collision energy (NCE) of 65%. System control and data processing were performed using the Xcalibur 2.2 (Thermo Electron, San Jose, CA, USA) software.

### 2.4. Cells and Culture Conditions

The following cell strains were used in this study: human neonatal foreskin fibroblasts AG01523 (obtained from Coriell Institute for Medical Research, Camden, NJ, USA), human neonatal foreskin fibroblasts BJ (obtained from the American Tissue Culture Collection ATCC, Rockville, MD, USA), human adult primary dermal fibroblasts DSF (from the pre-existing cell bank of the Laboratory of Cell Proliferation and Ageing, NCSR “Demokritos” [20]), human keratinocytes HaCaT (kindly provided by Prof. Evangelos Kolettas), and mouse melanoma B16-F10 cells (obtained from ATCC). Cells were cultured in Dulbecco’s Modified Eagle Medium (DMEM) supplemented with penicillin (100 U/mL)/streptomycin (100 mg/mL) (obtained from Biosera, Nuaillé, France) and 10% (*v*/*v*) FBS (from Life Technologies Europe BV Thessaloniki, Greece) in a humidified atmosphere of 5% CO_2_ at 37 °C and they were subcultured using a trypsin/citrate (0.25%:0.30% *w*/*v*) solution. Cell culture samples were periodically tested and found to be free of mycoplasma.

### 2.5. Assessment of Cell Viability

Cells (human skin fibroblasts and human keratinocytes) were plated in 96-well flat-bottomed transparent microplates (Corning Inc., Corning, NY, USA) at a subculture split ratio in DMEM 10% FBS. Following overnight incubation to ensure proper cell attachment, additional medium was added containing serial dilutions of the test extracts. Since the test extracts were dissolved in dimethylsulfoxide (DMSO; Sigma, St. Louis, MO, USA), corresponding dilutions of DMSO were used as controls. After incubation for 72 h, the medium was replaced by serum-free, phenol-red-free DMEM (PAN Biotech GmbH, Aidenbach, Germany) containing 1 mg/mL of 3-(4,5-dimethylthiazol-2-yl)-2,5-diphenyltetrazolium bromide (MTT; Sigma), and incubation was continued for 4 h. The medium was then discarded, the insoluble purple formazan crystals were solubilized in 2-propanol, and the absorbance was measured using a Spark multimode microplate reader equipped with plate stacker (Tecan Group Ltd., Männedorf, Switzerland) at a wavelength of 550 nm (reference wavelength 690 nm). Each experiment was performed in quadruplicate, and the results are the mean of three independent experiments. The results are presented in the form of the highest noncytotoxic concentrations detected, in order to be used in the subsequent cell-based assays.

### 2.6. Free-Radical Scavenging Assay

The ability of the extracts to scavenge the free radical 2,2-Diphenyl-1-Picrylhydrazyl (DPPH) was assessed as previously described [21]. Briefly, the extracts diluted in DMSO at their highest noncytotoxic concentration (see above Section 2.5) were thoroughly mixed with an equal volume of a fresh DPPH (Sigma) ethanolic solution (1 mM). The absorbance at 520 nm was measured at regular time intervals in a Tecan Spark multimode microplate reader. DMSO served as negative control, while the water-soluble vitamin E analogue 6-hydroxy-2,5,7,8-tetramethylchroman-2-carboxylic acid (Trolox; Sigma) at 100 μM was used as positive control.

### 2.7. Assessment of Intracellular Reactive Oxygen Species (ROS)

Cells (human skin fibroblasts and human keratinocytes) were plated in clear-bottomed black 96-well microplates (Porvair Ltd., Fareham, UK) at a 1:2 split ratio density in the presence of FBS. When the cells reached confluency, the test extracts were added, dissolved in serum-free medium at the indicated concentrations. DMSO at corresponding dilutions served as negative control, with Trolox (100 μM) and N-acetyl-cysteine (NAC; 5 mM; Sigma) as positive control for fibroblasts and keratinocytes, respectively. Following an overnight incubation, dichloro-dihydro-fluorescein diacetate (DCFH-DA; Sigma) was added at a final concentration of 10 μM in serum free medium and fluorescence emission at 520 nm (excitation wavelength: 485 nm) was measured after 24 h, using a Spark (Tecan) microplate reader. The experiments were repeated three times. Each time, the samples were added in quadruplicates. Furthermore, to assess the impact of selected test extracts on stimulated intracellular ROS levels, following overnight incubation with the extract, 200 μM H_2_O_2_ was added along with 5-(6)-chloromethyl-2′,7′-dichlorodihydrofluorescein diacetate acetyl ester (CM-H_2_DCFDA; Thermo Fisher Scientific Inc., Waltham, MA, USA) at 10 μM final concentration. After incubation for 30 min in the dark (37 °C), cells were lysed [150 mM NaCl, 1% (*v*/*v*) NP-40, 50 mM Tris, pH 8.0] and their protein content was measured using the Bradford assay (Bio-Rad Laboratories, Inc., Hercules, CA, USA). The produced fluorescence was measured in a Spark multimode microplate reader (Tecan) (490 nm excitation and 540 nm emission wavelengths).

### 2.8. Assessment of Photoprotective Activity

Cells (human skin fibroblasts and human keratinocytes) were plated in 96-well microplates at a density of 8000 cells/well in DMEM containing 10% (*v*/*v*) FBS. When the cells reached confluency, the test extracts diluted in DMEM 10% FBS were added at their highest non-cytotoxic concentration and at a 5-fold lower concentration. Following incubation for 5 h, the medium was discarded and replaced by PBS containing the same extract concentrations and the cells were placed in a UV box, as reported previously [22], under a UVB lamp (Sankyo Denki Co., Hiratsuka, Japan) with an emission spectrum of 280–360 nm and a peak at 306 nm. Fibroblasts and keratinocytes were irradiated for the appropriate time interval so as to receive a total energy of 420 mJ/cm^2^ and 700 mJ/cm^2^, respectively. After irradiation, medium was changed once more to DMEM 10% FBS containing the same extract concentrations, and the cells were incubated for 72 h. Cells treated with the corresponding DMSO concentrations and subjected to the same procedure but not exposed to the UV lamp served as controls. Cell viability at the end of the incubation time was assessed using the Neutral Red method, i.e., culture medium was replaced with serum-free, phenol-red-free DMEM containing neutral red (Sigma) at a concentration of 0.0075% (*w*/*v*). The uptake of the neutral red dye by viable cells was determined 4 h later, after washing with PBS and dissolving with a solution of ethanol and water (1:1) acidified with 1% acetic acid. The neutral red fluorescence emission was measured at 645 nm following excitation at 530 nm using a Spark multimode microplate reader (Tecan). The results are presented as percent inhibition of the cytotoxic activity exerted on DMSO-treated, UVB-treated cultures based on the following formula:

%Inhibition = (1 − ((NC − S)/(NC − UC)) × 100,

where S: UVB-treated sample, NC: non-UVB-treated control, and UC: UVB-treated control. Experiments were performed in quadruplicate, and the results presented are the mean of three independent experiments.

### 2.9. Inhibition of Enzymatic Activities

#### 2.9.1. Collagenase

To determine the effect of the extracts on collagenase activity, the fluorogenic substrate Dabcyl-Gaba-Pro-Gln-Gly-Leu-Glu (EDANS)-Ala-Lys-NH2 (TNO211; Cayman Chemical, Ann Arbor, MI, USA) was used [23]. Extracts at their highest non-cytotoxic concentration were mixed with *Clostridium histolyticum*-derived collagenase (Sigma) diluted in Tris-HCl buffer supplemented with Ca^2+^ (pH: 7.5) at a concentration of 50 μg/mL and incubated for 15 min, at 37 °C, in black 96-well plates (Greiner Bio-One GmbH, Kremsmünster, Austria). TNO211 was then added at a final concentration of 10 μM and the fluorescence emission was monitored at 480 nm, following excitation at 340 nm, using a Tecan Spark multimode microplate reader. Vehicle (DMSO) and epigallocatechin gallate (EGCG) were used as negative and positive control, respectively [24]. The fluorescence of a blank sample, containing the buffer and the substrate but no enzyme, was also measured. The percentage of collagenase activity was calculated with the following equation:

%Collagenase activity = ((S − B)/(C − B)) × 100,

where S: sample, B: blank, and C: control. The experiment was conducted twice, and each time the samples were added in duplicates. The results are demonstrated as mean ± SD.

The above method was also adapted to measure matrix metalloproteinase (MMP) activity secreted by human skin fibroblasts. Cells were plated in 12-well tissue cultures plates and cultured until confluency. Then, the culture medium was replaced by serum-free DMEM containing the test extracts, each at its highest non-cytotoxic concentration. After incubation for 24 h, all wells received serum-free DMEM and were further incubated for 24 h. Conditioned media (CM) were collected from all wells, clarified by centrifugation (10,000× *g*, 10 min, 4 °C), and incubated with TNO211 (10 μM). Fluorescence was measured at 480 nm (excitation wavelength 340 nm), as described above. Cultures treated with the corresponding DMSO concentration yielded the control CM.

#### 2.9.2. Elastase

To determine the effect of the extracts on elastase activity, the substrate N-(methoxysuccinyl)-L-alanyl-L-alanyl-L-prolyl-L-valine 4-nitroanilide (Sigma) was used. The test extracts at their highest non-cytotoxic concentration were pre-incubated with 5 mM of the above elastase substrate in a Tris-HCl buffer (0.2 M, pH: 8.0) for 15 min at 37 °C. Then, elastase from porcine pancreas (Sigma; 0.3 U/mL) was added and the mixture was further incubated for 15 min at 37 °C, before measurement of the absorbance at 495 nm in a Tecan Spark multimode microplate reader. Vehicle (DMSO) and N-(methoxysuccinyl)-L-alanyl-L-alanyl-L-prolyl-L-valine chloromethylketone (Sigma) at 750 μM were used as negative and positive control, respectively. The absorbance of a blank sample, containing the buffer and the substrate but no enzyme, was also measured. Percentage of the enzyme inhibition was calculated according to the following equation:

%Inhibition = (((C − Cb) − (S − Sb))/(C − Cb)) × 100,

where C: control, Cb: blank control, S: sample, Sb: blank sample. Each sample was tested in duplicate, and the results are presented as mean ± SD of two independent experiments.

The above method was also adapted to measure elastase activity in cell lysates of human skin fibroblasts. Confluent cell cultures were incubated with selected extracts at the indicated concentrations for 24 h. Then, the cells were trypsinized, washed in PBS, collected by centrifugation (500× *g*, 5 min, room temperature), and lysed in 20 mM Tris HCl (pH 7.6) containing 20 mM KCl, 1 mM EDTA, 1 mM dithiothreitol, 0.2% (*v*/*v*) Nonidet P 40, 5 mM ATP, and 10% (*v*/*v*) glycerol. Protein content of the lysates was assessed using the Bradford method, and 30 μg protein were used to assess the ability of each lysate to inhibit porcine pancreas elastase activity, as described above.

#### 2.9.3. Tyrosinase

Inhibition of mushroom tyrosinase activity was assessed using L-DOPA as substrate based on the absorbance of the dopachrome formed [16]. The tested extracts diluted in phosphate buffer (0.1 M KH_2_PO_4_/K_2_HPO_4_, pH 6.5) were incubated with a mushroom tyrosinase (Sigma) solution (100 U/mL) in a 96-well microplate at room temperature for 10 min in the dark. Then, L-DOPA (Merck KGaA, Darmstadt, Germany) was added at a concentration of 2.5 mM, and absorbance at 475 nm was recorded using a Tecan Spark multimode microplate reader. Each extract was used at its highest non-cytotoxic concentration. Vehicle (DMSO) and kojic acid (Apollo Scientific, Stockport, UK) served as negative and positive control, respectively. A blank for each extract (including controls) was studied in parallel, in the absence of tyrosinase. Percentage of the enzyme inhibition was calculated according to the following equation:

%Inhibition = (((C − Cb) − (S − Sb))/(C − Cb)) × 100,

where C: control, Cb: blank control, S: sample, Sb: blank sample. Each sample was tested in duplicate, and the results are presented as mean ± SD of two independent experiments.

In order to assess tyrosinase activity in cell lysates, B16F10 cells were plated in 6-well plates for 24 h before their incubation with the indicated concentrations of the tested extracts for another 24 h. Then, the cells were trypsinized, washed in PBS, collected by centrifugation (500× *g*, 5 min, room temperature), and lysed with 1% NP-40 lysis buffer (pH 6.8). An amount of 20 μg of total protein was diluted in PBS and mixed with 5 mM L-DOPA (Merck KGaA, Darmstadt, Germany). Samples were set in triplicate in a 96-well plate and were incubated at 37 °C for 1 h. The absorbance was measured at 492 nm using a Tecan Spark microplate reader. Kojic acid was used as positive control.

### 2.10. Inhibition of Melanogenesis in Zebrafish

#### 2.10.1. Zebrafish Maintenance and Breeding

Zebrafish embryos were raised under standard laboratory conditions at 28 °C [25]. The genetic backgrounds used were wild-type AB strains for all the screenings. Zebrafish were maintained in accordance with the European Directive 2010/63 for the protection of animals used for scientific purposes and the Recommended Guidelines for Zebrafish Husbandry Conditions. The experimental protocols described in this study were carried out with zebrafish larvae up to 96 h post-fertilization (hpf), and therefore these experiments are not considered animal experiments and do not fall under the protection guidelines of the directive 2010/63/EU revising directive 86/609/EEC on the protection of animals used for scientific purposes as adopted on 22 September 2010.

#### 2.10.2. Compound Treatment and Phenotype-Based Evaluation of Melanogenesis

For the calculation of LC_50_, the OECD guidelines for Fish Embryo Acute Toxicity Test (TG 236) were used [26]. In brief, newly fertilized zebrafish eggs were exposed to the test compounds at 3 hpf for a period of 96 h. Every 24 h, lethal effects were assessed and compared to the control values. At the end of the exposure period, acute toxicity was determined and LC_50_ was calculated. For monitoring the melanogenic inhibitory activity, synchronized and dechorionated 24 hpf embryos were treated with various concentrations of the extracts (below the LC_50_) in 12-well plates containing 2 mL of embryo medium (0.3 g/L “Instant Ocean” Sea Salts and 0.08 g/L CaSO_4_⋅2H_2_O). Test compounds were dissolved in DMSO that was always used as vehicle control up to 0.1% (*v*/*v*) final concentration. In all experiments, 0.2 mM 1-phenyl-2-thiourea (PTU), a known TYR inhibitor, was considered as a standard positive control. The testing period varied between 24 and 48 h and the effects on the pigmentation were observed under a stereomicroscope.

### 2.11. Effects on Proteasomal and Lysosomal Activities

#### 2.11.1. Proteasomal Activity

For measuring proteasome peptidase activity, human skin fibroblasts and human keratinocytes, treated with the tested extracts at the indicated concentrations for 24 h, were lysed on ice in a buffer suitable for the isolation of intact 26S proteasomes (0.2% NP-40, 5 mM ATP, 10% glycerol, 20 mM KCl, 1 mM EDTA, 1 mM dithiothreitol, and 20 mM Tris, pH 7.6). Lysates were cleared by centrifugation at 19,000× *g* (4 °C) and protein content was estimated with the Bradford assay (Bio-Rad, Hercules CA, USA). Cleared supernatants were immediately used to determine the chymotrypsin-like (CT-L/LLVY) proteasome proteolytic activity by a 30 min incubation with the fluorogenic peptide Suc-Leu-Leu-Val-Tyr-AMC, (Enzo Life Sciences Inc., Farmingdale, NY, USA) at 37 °C. Fluorescence was recorded in a Tecan Spark microplate reader at excitation and emission wavelengths of 350 nm and 440 nm, respectively.

#### 2.11.2. Lysosomal Activity

For measuring cathepsins’ activity, human skin fibroblasts and human keratino-cytes, treated with the tested extracts at the indicated concentrations for 24 h, were lysed in an extraction buffer (1 mM dithiothreitol and Tris 50 mM, pH 4.0) and the lysates were cleared at 17,000× *g* for 20 min at 4 °C. Following protein content measurement with the Bradford assay, 5 μg of protein was incubated in the reaction buffer (50 mM sodium acetate, 8 mM cysteine-hydrochloride, 1 mM EDTA, pH 4.0) containing the fluorogenic substrate Z-FR-AMC (Enzo Life Sciences Inc.) for 30 min at 37 °C. Fluorescence was measured in a Spark multimode microplate reader (Tecan) at excitation and emission wavelengths of 350 and 440 nm, respectively.

### 2.12. Assessment of Gene Expression

Confluent cultures of human skin fibroblasts were incubated with selected extracts at the indicated concentrations for 24 h. Then, total RNA was extracted using RNAiso plus reagent (DSS Takara Bio India LTD, New Delhi, India) and quantified in a BioSpec nano spectrophotometer (Shimadzu, Kyoto, Japan). cDNA was produced from 1 μg of RNA using the FastGene Scriptase II cDNA Kit (NIPPON Genetics, Düren, Germany), according to the manufacturer’s instructions, and subjected to qPCR reactions using the HOT FIREPol^®^ EvaGreen^®^ qPCR Supermix (Solis BioDyne, Tartu, Estonia) in a PikoReal^TM^ Real-Time PCR System (Thermo Fisher Scientific Inc., Waltham, MA, USA). mRNA levels were quantified in comparison to the untreated control using the 2^−ΔΔCT^ method [27] and glyceraldehyde-3-phosphate dehydrogenase (GAPDH) as the reference gene. Sequences of the primers used in the current study are presented in Supplementary Table S1.

## 3. Results

### 3.1. Plant Material and Assessment of Cytotoxicity

Greece is widely acknowledged as a biodiversity hotspot, home to numerous native plant species with potent therapeutic properties. These species hold significant value for industries such as cosmetics, pharmaceuticals, and food [16]. Therefore, based on prior research and ethnobotanical knowledge, the plant species studied were selected from families including, among others, *Asteraceae*, *Fabaceae*, *Lamiaceae*, *Ericaceae*, and *Onagraceae* (Table 1). The selection of plants was based on previous knowledge related to cosmetic applications, as well as literature data. To comprehensively analyze the composition of the collected plants, each sample was dried, pulverized, and extracted using two environmentally sustainable methods: SFE-CO_2_ and ASE. These methods are known for their efficiency in recovering a wide range of metabolites with different polarities, making the extracts suitable for diverse industrial applications [28]. ASE predominantly yielded medium- to high-polarity compounds, with extraction efficiencies ranging from 53.5% to 5.54% (*w*/*w*), while SFE was more effective for non-polar compounds, with yields ranging from 16.6% to 0.63% (*w*/*w*).

All extracts were subjected to cytotoxicity assessment, both on human skin fibroblast cultures and keratinocytes, using the MTT method. Concentrations tested were from 0.032 μg/mL to 100 μg/mL. In Table 2, the highest non-cytotoxic concentration for each extract is depicted. This concentration was further used in all experiments.

### 3.2. Antioxidant Activity

#### 3.2.1. Radical Scavenging Activity

The ability of the extracts at their highest non-cytotoxic concentration to scavenge the free radical DPPH is presented in Table 3. The most active extract was found to be 7A achieving a DPPH inhibition similar to that of the positive control. In general, extracts obtained through ASE (with the suffix A) were found to be more active than the ones obtained through SFE (with the suffix B).

#### 3.2.2. Attenuation of ROS

Beyond the assessment of free radical scavenging in a cell-free system (see above DPPH), the ability of the extracts to suppress ROS intracellularly was evaluated in human skin fibroblast and human keratinocyte cultures (Table 4A and 4B, respectively). A total of 34 extracts were found to be equally or more potent than the positive control (Trolox at 100 μM) in fibroblast cultures. In general, the extracts seemed to be less efficient in attenuating ROS levels in keratinocyte cultures.

**Figure 1:**
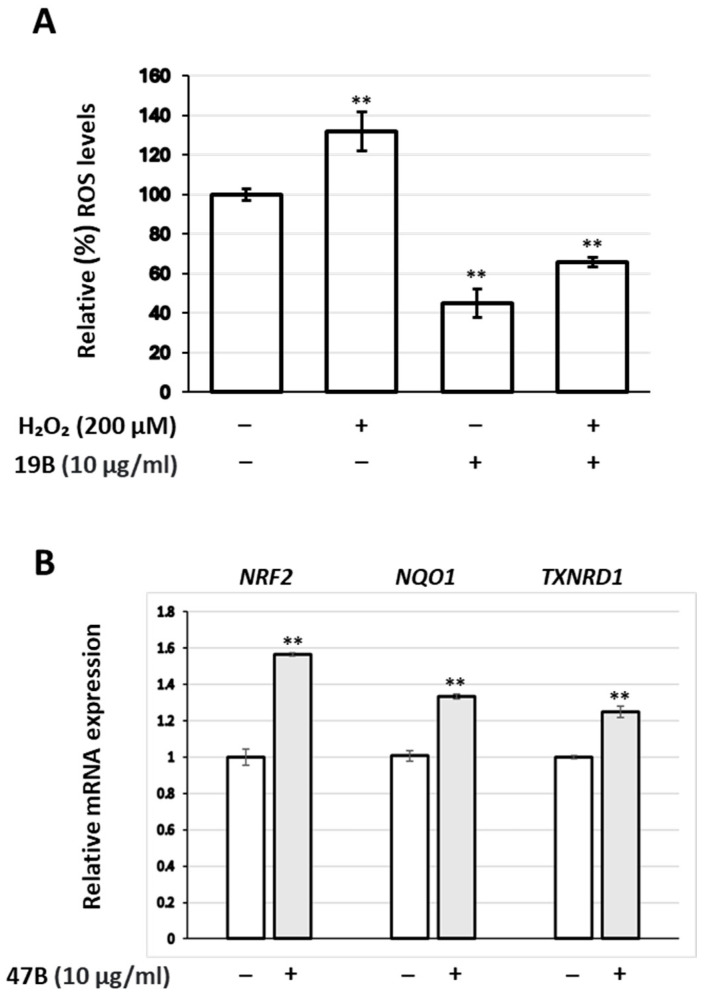
(**A**) Relative (%) ROS levels of human skin fibroblasts treated with 19B at 10 μg/mL and/or H_2_O_2_ (200 μM) for 24 h. (**B**) Relative expression levels of the *NRF2* gene, as well as its downstream targets (*NQO1* and *TXNRD1*), in human skin fibroblasts treated with the extract 47B at 10 μg/mL for 24 h. Values in control samples were set to 100% (**A**) or 1 (**B**); GAPDH gene expression was used as a reference for total RNA input. Bars, ±SD; ** *p* < 0.01.

Results presented in Table 4 refer to the basal intracellular ROS levels. An extract (19B) was randomly selected among the active ones for testing its capacity to counteract the effect of the oxidative stress-inducing factor, H_2_O_2_ (200 μM), in human skin fibroblasts. Indeed, 19B at 10 μg/mL was found capable to suppress not only basal intracellular ROS levels but also intracellular ROS stimulated by H_2_O_2_ (Figure 1A). Moreover, incubation of fibroblasts with the (randomly selected) extract 47B at 10 μg/mL concentration led to a mild up-regulation of the *NRF2* gene, as well as its downstream targets *NQO1* and *TXNRD1* (Figure 1B).

### 3.3. Photoprotection

Photoprotective abilities of the extracts were assessed by exposure of both human skin fibroblast (DSF) and keratinocyte (HaCaT) cultures to the extracts’ highest non-cytotoxic concentrations prior to, during, and post irradiation with UVB doses known to lead to cell death at approx. 50% compared to the non-irradiated control. As shown in Table 5, the highest photoprotection to DSF cells was conferred by extracts 8A and 40A, while 3A, 5A, 11A, and 21A were also active to a lesser extent. In general, the extracts were found to be less active on HaCaT cell cultures.

### 3.4. Inhibition of Enzymatic Activities Related to Skin Health

#### 3.4.1. Collagenase Inhibition

The ability of the extracts to inhibit enzyme activities with crucial roles for skin health, i.e., collagenase, elastase, and tyrosinase was assessed in cell-free systems, and the results are shown in Table 5, Table 6, and Table 7, respectively. A total of 17 extracts, all belonging to the ASE group, were found to inhibit collagenase activity, the most active (inhibition over 25%) being 7A, 20A, 21A, 3A, 5A, 8A, and 51A (Table 6).

**Figure 2:**
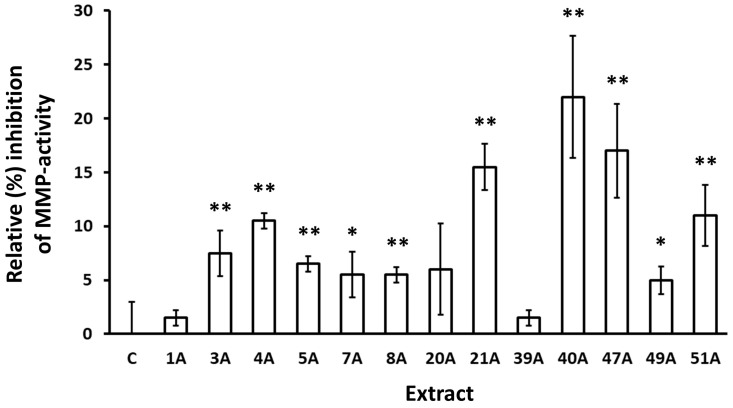
Inhibition of MMP activity secreted by human skin fibroblasts following incubation with the indicated extracts for 24 h. Control (conditioned medium from cultures treated with vehicle) was set to 0%. Bars, ±SD; * *p* < 0.05; ** *p* < 0.01.

To test whether this activity is retained also against human skin cell-produced collagenases, MMP activity secreted by human skin fibroblasts was also measured following incubation for 24 h with selected extracts. As shown in Figure 2, the activity of these extracts against fibroblast-secreted MMPs was confirmed.

#### 3.4.2. Elastase Inhibition

**Figure 3:**
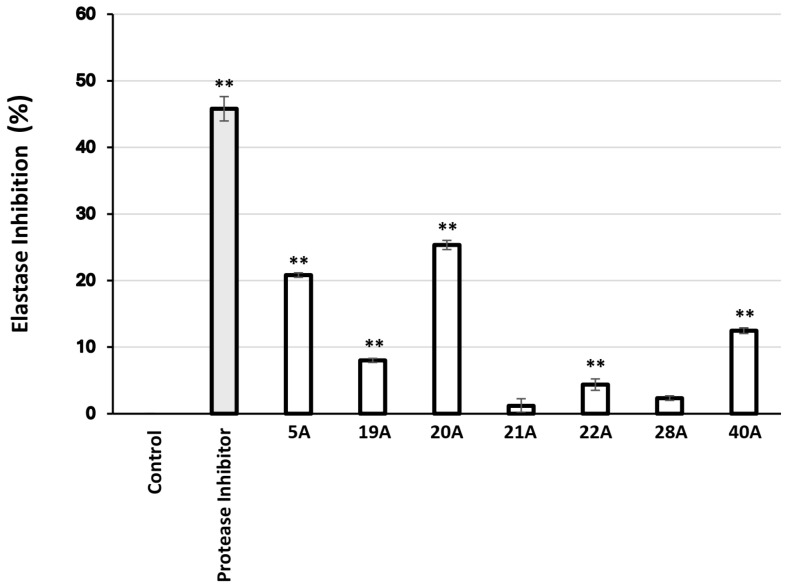
Relative (%) elastase inhibitory activity in human skin fibroblast cultures after 24 h of treatment with 10 μg/mL of the extracts. Protease inhibitor at 10 μL/mL was used as a positive control. Bars, ± SD; ** *p* < 0.01.

Moreover, 27 extracts were found to inhibit elastase activity (Table 7), the most active being 20A, 21A, 5A, 22A, 28, and 19A. The most promising extracts were then examined for their effect on elastase activity in human skin fibroblast cultures. As observed in Figure 3, the extracts 5A and 20A were found to exhibit significant anti-elastase activity.

#### 3.4.3. Tyrosinase Inhibition

Finally, 93 extracts were found to inhibit mushroom tyrosinase activity (Table 8), the most active (inhibition > 50%) being 10B, 20A, 30A, 22A, 21A, 37A, 1A, 37B, 5A, 40A, and 21B.

**Figure 4:**
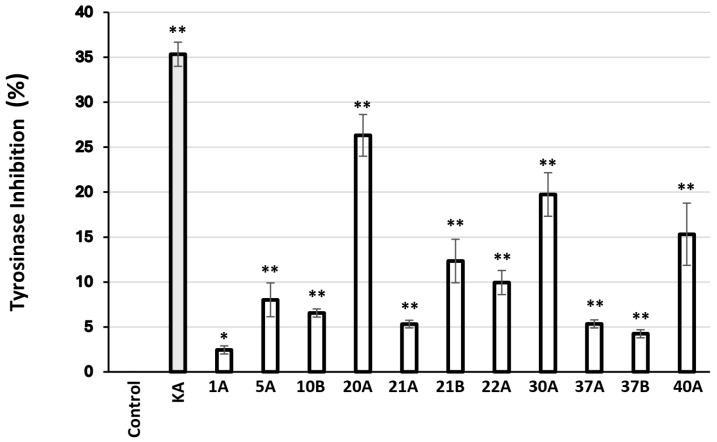
Relative (%) tyrosinase inhibitory activity in murine B16F10 melanocytes after 24 h of treatment with 10 μg/mL of the extracts. Kojic acid (KA) at 2 mM was used as a positive control. Bars, ± SD; * *p* < 0.05; ** *p* < 0.01.

These most active extracts identified *in vitro* were subsequently evaluated for their biological activity in B16F10 melanocytes to confirm their efficacy (Figure 4).

#### 3.4.4. *In Vivo* Evaluation of Tyrosinase Inhibition Using a Zebrafish Embryo Melanogenesis Assay

**Figure 5:**
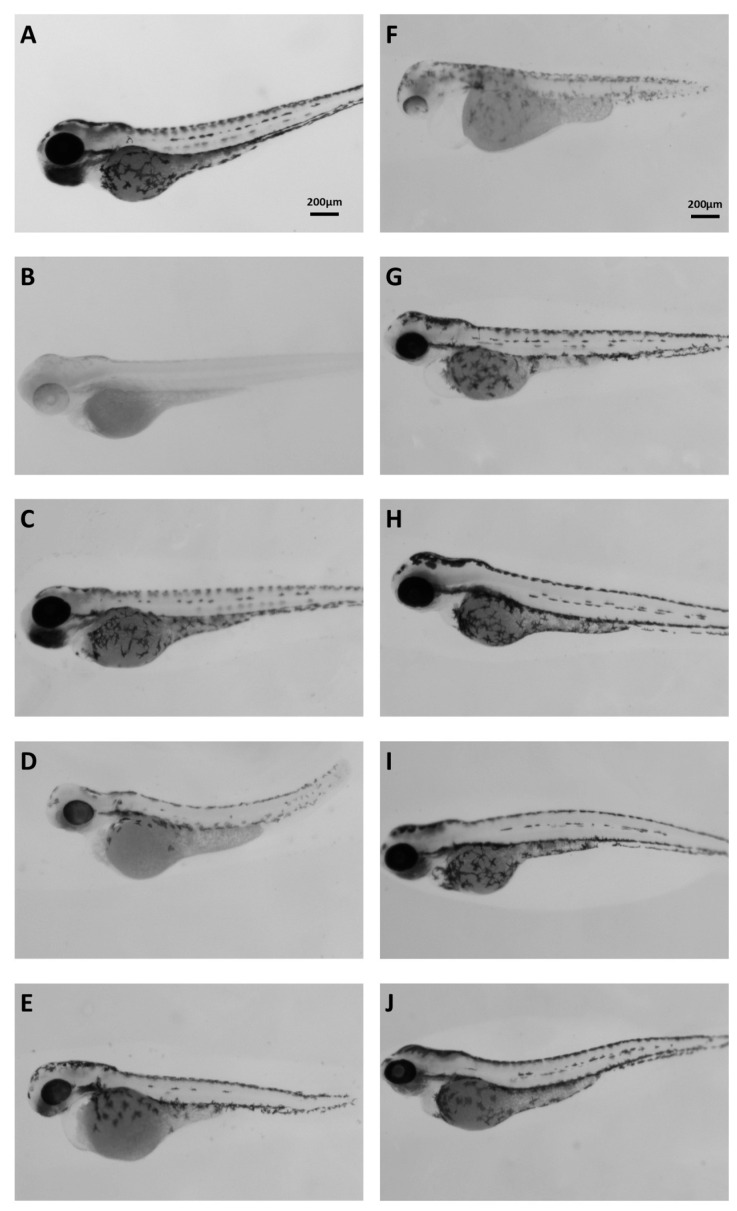
Brightfield images of 72 hpf embryos. (**A**) Control; (**B**) PTU-treated embryos; (**C**) 1A 200 μg/mL; (**D**) 10B 20 μg/mL; (**E**) 20A 20 μg/mL; (**F**) 22A 100 μg/mL; (**G**) 21A 100 μg/mL; (**H**) 30A 100 μg/mL; (**I**) 37A 200 μg/mL; (**J**) 37B 100 μg/mL.

Extracts were tested *in vivo* by adding them in the water of developing zebrafish embryos. According to the guidelines for fish embryo toxicity test (OECD 236), we estimated the LC_50_ and then went on and retested for more specific phenotypes at different (including sublethal) concentrations. Several morphological phenotypes were noted but focusing on the inhibition of melanocyte differentiation or melanin synthesis assay [29], we identified the following extracts that were also active in the *in vitro* tyrosinase inhibition assay: 10B (Figure 5D), 20A (Figure 5E), and 22A (Figure 5F) were the most potent, followed by 21A (Figure 5G). In addition, 1A, 30A, 37A, and 37B also inhibited melanogenesis but showed less activity *in vivo* (Figure 5). As a positive control, in this assay, the commonly used 1-phenyl 2-thiourea (PTU) was employed. In this figure, the extracts were added 24 h post-fertilization (hpf) and embryos were dechorionated by pronase treatment. This methodology was utilized in the whole extract samples in order to bypass early developmental defects and putative difficulties of the relevant bioactive compounds to penetrate the chorion of zebrafish embryos but still use the highest possible concentration.

### 3.5. Effect of the Extracts on the Main Proteostatic Pathways

**Figure 6:**
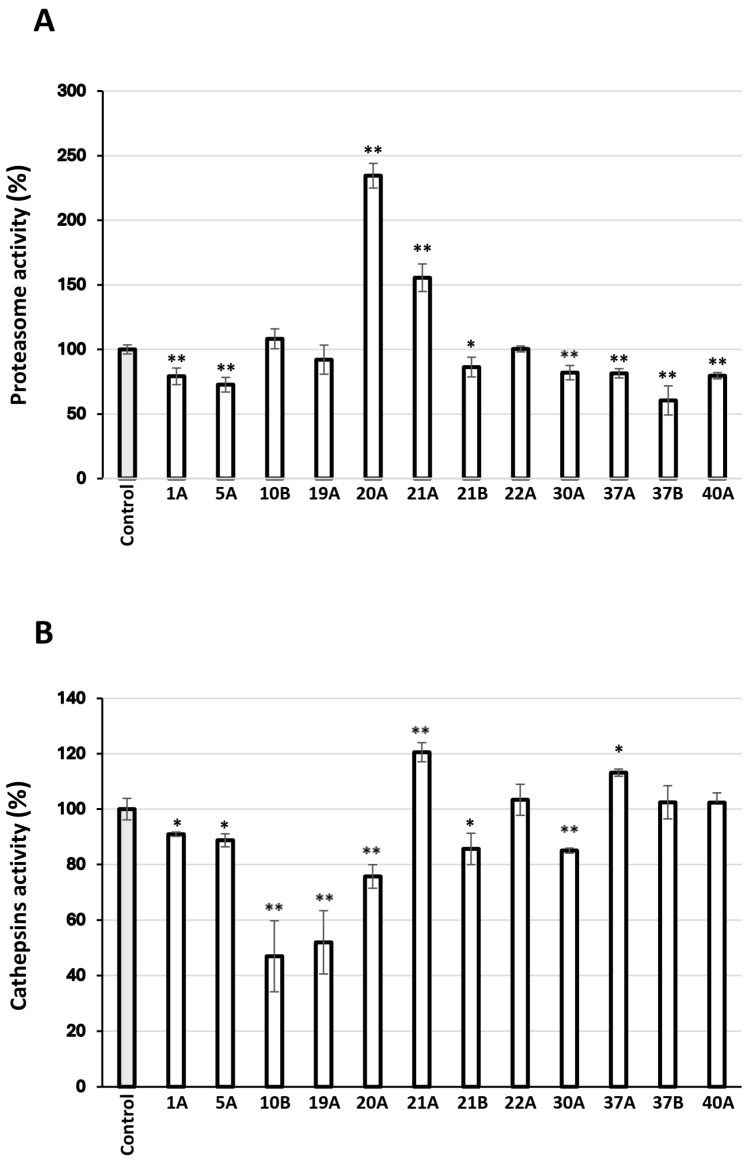
(**A**) Relative (%) chymotrypsin-like proteasome peptidase activity in human skin fibroblasts treated with 10 μg/mL of the extracts for 24 h. (**B**) Relative (%) lysosomal-cathepsins activity in human skin fibroblasts treated with 10 μg/mL of the extracts for 24 h. Control samples values were set to 100%. Bars, ± SD; * *p* < 0.05; ** *p* < 0.01.

The most promising extracts were further examined for their capacity to activate cellular protective mechanisms, which ensure proteome stability and integrity. Specifically, their effects on the main proteostatic pathways were investigated to assess their potential cytoprotective role. We observed that the extracts 20A and 21A increased the main proteasome peptidase activity, i.e., the chymotrypsin-like activity (CT-L/β5) in human skin fibroblasts (Figure 6A). Furthermore, cell exposure to the extract 21A at the concentration of 10 μg/mL led to significant induction of cathepsins enzymatic activity (Figure 6B).

## 4. Discussion

The aim of the present work was to evaluate the chemical content and the biological properties of a great variety of Greek plants towards their potential use in skin protection applications. The plants belonged to 20 different families, i.e., *Adoxaceae*, *Anacardiaceae*, *Apiaceae*, *Asteraceae*, *Boraginaceae*, *Cactaceae*, *Cistaceae*, *Crassulaceae*, *Cupressaceae*, *Ericaceae*, *Fabaceae*, *Hypericaceae*, *Lamiaceae*, *Malvaceae*, *Onagraceae*, *Paeoniaceae*, *Pinaceae*, *Plumbaginaceae*, *Polygonaceae*, and *Rutaceae*. Extraction of each plant material was performed using two different techniques, i.e., ASE and SFE-CO_2_, both being environmentally friendly, fast, cost-effective, and providing fully controllable conditions and hence extracts with consistent compositions [30]. The initial step of the extracts’ biological assessment was the evaluation of their cytotoxicity on human skin fibroblasts and keratinocytes. As shown in Table 2, most of the extracts were not cytotoxic at the highest concentration tested (100 μg/mL) following a 72-hr incubation (61 extracts for fibroblasts and 70 extracts for keratinocytes). The highest non-cytotoxic concentration of 20 μg/mL was determined for 30 extracts regarding fibroblasts and for 23 extracts regarding keratinocytes, while 12 extracts (regarding fibroblasts) and 11 extracts (regarding keratinocytes) exhibited lower limits of cytotoxicity. All subsequent *in vitro* assays (even cell-free ones) were performed at these highest non-cytotoxic concentrations for each extract, ensuring the applicability of our findings on living skin cells. 

All extracts were assessed for their antioxidant capacity by means of (a) a cell-free assay (DPPH) and (b) their ability to suppress intracellular ROS levels in human skin fibroblast and keratinocyte cultures. The most active extracts in the cell-free assay were those obtained through ASE from *Cistus creticus* ssp. *eriocephalus* and *Polygonum idaeum*, suppressing DPPH absorbance to 13.2% and 15.1% of the vehicle absorbance, respectively (Table 3), hence being more active than Trolox (100 μM), which was used as a positive control. An additional 19 extracts were also active to a lesser extent (between 20% and 50% of the vehicle absorbance), 54 were less active yet showed DPPH absorbance below that of the vehicle, and the remaining 29 were inactive. Interestingly, the extracts produced through ASE (which yields extracts with more polar constituents [31]) were more active, in agreement with previous studies showing higher antioxidant capacity in polar than non-polar extracts [32,33,34]. The existing literature supports our findings for many of the plants identified as possessing significant antioxidant activity, such as *C. creticus*, *C. salvifolius*, *C. parviflorus* [35], *E. dodonaei* [36], *S. pomifera* [37], *S. sediforme* [38], *E. parviflorum* [39], *S. officinalis* [40,41], *C. siliqua* [21], *H. perforatum* [42], *J. oxycedrus* [43], *S. Sclarea* [41], *J. turbinata* [44], *C. siliquastrum* [45], *C. coggygria* [46], *A. unedo* [47], and *P. terebinthus* [48], while for some other plants, e.g., *P. idaeum*, *A. graecum*, *A. cephalonica*, and *A. canencens*, an antioxidant activity is reported here for the first time.

**Figure 7:**
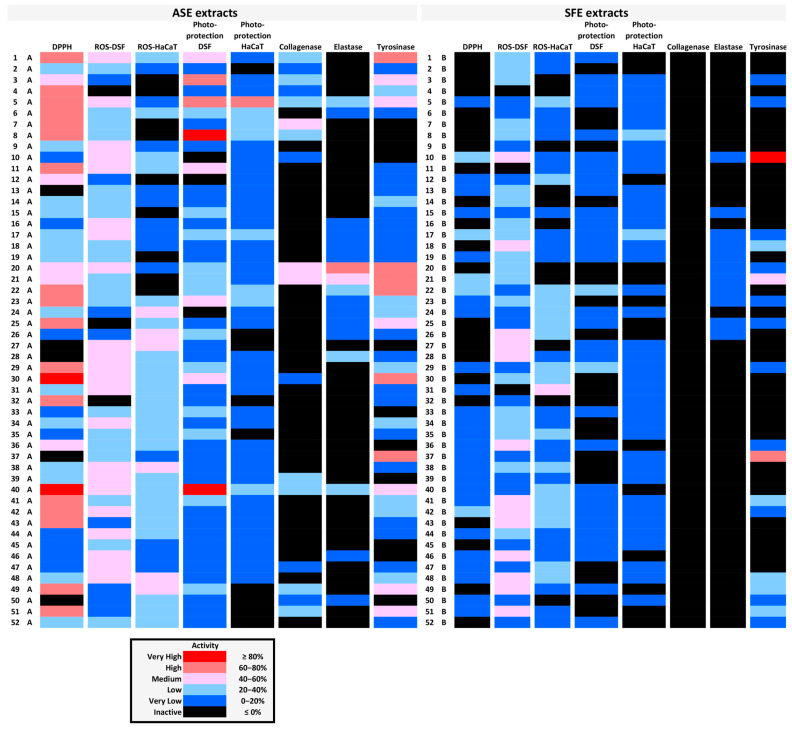
Graphic representation of the various biological activities of each extract in the form of a heatmap. Inhibition of the activities presented in Table 2, Table 3, Table 4, Table 5, Table 6 and Table 7 (see above) is depicted here in different colors depending on its degree. The bioactivities of ASE vs. SFE extracts of each plant material are shown in each horizontal line.

Antioxidant activities of the extracts as estimated by the reduction of intracellular ROS levels using the DCFH-DA assay were generally qualitatively different from those obtained through the DPPH assay (compare Table 3 and Table 4; Figure 7). In most of the cases, the extracts exhibited higher activity in the DCFH-DA assay than in the DPPH one (72 out of 104 extracts for fibroblast cultures; 60 out of 104 for keratinocytes). Moreover, 90% of the 32 extracts exhibiting higher DPPH inhibition than ROS suppression in fibroblast cultures was obtained through ASE, similarly to 75% of the 44 extracts showing higher activity in the DPPH assay than ROS suppression in keratinocytes. This could be the outcome of the extracts’ different capacity for cell internalization, which may depend on their polarity, among others. On the other hand, such differences may stem from the inherent limitations of each assay [49]. In any case, live cell assays for determining the antioxidant activity of plant extracts are considered to be a closer simulation of the *in vivo* responses [50]. Moreover, ROS suppression was more effective in fibroblast cultures than in keratinocytes (80 out of 104 extracts were more potent for fibroblasts), which may be the outcome of different basal ROS levels between the two cell types. Nevertheless, extract 19B from *Origanum vulgare* ssp. *hirtum* was found to also be able to suppress external induction of ROS levels by hydrogen peroxide treatment (Figure 1A). In agreement with this observation, an ethanolic *O. vulgare* extract has been reported to inhibit hydrogen peroxide cytotoxicity against human A549 cells [51]. Assessment of the extract 47B (from *Citrus medica*) led to the observation that ROS suppression in human skin fibroblasts is accompanied by activation of the NRF2 transcription factor, as well as its downstream targets NQO1 and TXNRD1 (Figure 1B). These results agree with reports on NRF2 activation by various components of *Citrus* plants [52,53,54,55,56,57].

Regarding photoprotection of human skin fibroblasts, the most active extracts were those obtained through ASE from *Epilobium parviflorum* and *Polygonum idaeum* (exhibiting inhibition of UVB-induced cytotoxicity by 88% and 86%, respectively), followed by *Cistus parviflorus* (64%), *Arbutus unedo* (62%), *Hypericum perforatum* (56%), *Cistus creticus* ssp. *eriocephalus* (54%), *Acantholimon graecum* (48%), *Pistacia terebinthus* (43%), *Abies cephalonica* (41%), and others with less than 40% inhibitory activity (Table 5). A previous study assessing photoprotection based on UV-induced hydroxyl radical generation failed to detect the photoprotective activity of *E. parviflorum* [58], while the protective activity of *P. idaeum* has also not been reported elsewhere. Besides the already known protection conferred by *A. unedo* against the cytotoxic effects of UVB irradiation [59] and similar reports on other subspecies of the genera *Cistus*, *Hypericum*, and *Pistacia* [60,61,62], extracts identified as photoprotective through the present screening represent novel findings. Interestingly, the photoprotection of human keratinocytes by these extracts was generally less pronounced (Table 5), with the exception of the ASE extract from *C. parviflorus* (65% inhibition of UVB-induced cytotoxicity), a fact that could be related to the higher survival rates of keratinocyte monolayers compared to fibroblasts following their exposure to UV radiation [63]. Moreover, for most of the plants studied here, ASE extracts exhibited more potent photoprotective activity than SPE ones, possibly indicating that this activity may be conferred by polar molecules [64].

During the assessment of the putative inhibitory capacity of the extracts against collagenase enzymatic activity, 17 out of 104 extracts were found to be active (Table 6). Interestingly, all of them were produced through ASE, while none of the SFE-derived extracts were active. The highest inhibition of collagenase activity was achieved by *Epilobium dodonaei* extract, followed by *Pistacia lentiscus*, *Pistacia terebinthus*, *Arbutus unedo*, *Cistus parviflorus*, *Epilobium parviflorum*, *Juniperus turbinata*, *Abies cephalonica*, *Paeonia mascula* ssp. *hellenica*, *Juniperus oxycedrus* ssp. *deltoides*, *Polygonum idaeum*, *Ceratonia siliqua*, and *Citrus medica*, and *Achillea millefolium*. *E*. *dodonei*, and *E*. *parviflorum* anti-collagenase activities are reported here for the first time, though there are similar reports in the literature regarding *Epilobium augustifolium* [65,66]. Regarding the extracts from the leaves and branches of the two *Pistacia* species, this is also a novel report, though we have previously shown the anti-collagenase activity of a *P. lentiscus* gum extract [67], and other teams have shown such an activity for other species of the *Pistacia* genus [68,69]. Inhibition of collagenase activity has also been reported previously for *A. unedo* [69] and *C. siliqua* [21]. All other extracts have not been tested previously for anti-collagenase activity, though data for other species of certain genera can be found in the literature, such as for *Paeonia lactiflora* [70], *Polygonum cuspidatum* [71], and *Achillea sintenisii* [72]. Beyond the inhibitory activity of the extracts against a collagenase of exogenous origin (from *Clostridium histolyticum*), their activity against MMPs produced by human skin fibroblast cultures was also studied and verified. All extracts tested were also found to inhibit fibroblast-secreted MMPs, although at different percentages from those observed against *C. histolyticum* collagenase (Figure 2). This may be due to differences among the activities of the different enzymes, but it may also reflect possible effects of the test extracts on MMP expression and secretion by the fibroblast cultures. More detailed analysis for each extract would be necessary to delineate these mechanisms. The most potent inhibition of fibroblast-MMP activity was achieved by *Polygonum idaeum* extract, followed by *Citrus medica* and *Pistacia terebinthus*. Notably, the inhibition of MMPs by *C. medica* extract aligns perfectly with published observations showing inhibition of MMP expression and/or activity by extracts from various *Citrus* species and isolated constituents *in vitro* and *in vivo* [73,74,75].

Assessment of the extracts’ ability to inhibit elastase activity yielded 27 active extracts out of 104 (Table 7). Again, ASE extracts exhibited higher activities than SFE ones, though—in contrast to collagenase inhibitory capacity—some elastase inhibitory activity could be recovered in SFE extracts in certain cases (e.g., *P. lentiscus* and *P. terebinthus*). The most active extracts in terms of elastase inhibition were those from *Pistacia lentiscus* and *Pistacia terebinthus*, followed by *Cistus parviflorus*, *Sedum sediforme*, *Polygonum idaeum*, *Atractylis gummifera* B, and *Origanum vulgare* ssp. *hirtum*. Our data agree with previous studies on extracts from plants of the *Pistacia* genus [68,76,77,78]. This is the first time that anti-elastase activity is shown in extracts from *C. parviflorus*, *S. sediforme*, *P. idaeum*, *A. gummifera*, and *O. vulgare* ssp. *hirtum*, although reports on other species of these genera exist in the literature, e.g., for a resin from *Cistus ladanifer* L. [79], for a leaf extract from *Sedum dendroideum* [80], for flavonol glucuronides from *Polygonum aviculare* L. [81], and for an *Origanum vulgare* L.-derived essential oil [82]. Beyond inhibition of porcine pancreas elastase in a cell-free assay system, the most active extracts were also used to treat human skin fibroblast cultures, and then the elastase-inhibitory activity of the cell lysates was determined (Figure 3). The results from the cell-based assessment are in line with those from the cell-free assay, confirming that the extracts are capable to exert their anti-elastase activity in the cellular environment.

Regarding inhibition of mushroom tyrosinase activity, the most active extracts were found to be those from *Glycyrrhiza glabra* (SFE), *Pistacia lentiscus* (ASE), *Cistus creticus* ssp. *eriocephalus* (ASE), *Sedum sediforme* (ASE), *Pistacia terebinthus* (ASE), *Hippocrepis emerus* (ASE), *Abies cephalonica* (ASE), *Hippocrepis emerus* (SFE), *Cistus parviflorus* (ASE), *Polygonum idaeum* (ASE), *Pistacia terebinthus* (SFE), *Juniperus turbinata* (ASE), *Juniperus oxycedrus* ssp. *deltoides* (ASE), and *Arbutus unedo* (ASE), in order of decreasing activity (Table 8). Most of these extracts were also found to inhibit statistically significantly melanogenesis in murine melanocytes *in vitro* (Figure 4). Moreover, we have used the zebrafish embryo melanocyte pattern and melanin quantification in order to identify and functionally verify the above tyrosinase-inhibiting extracts *in vivo* (Figure 5). Despite the complexity of the whole extracts, several active ones were identified and a phenotype-driven sub-fractionation would be necessary to enrich for the relevant bioactive compound. *G. glabra* extracts have been reported to inhibit tyrosinase activity and melanogenesis [83,84,85], most probably due to the presence of glabridin [83,86]. Interestingly, *G. glabra* extracts were also reported to contain other molecules capable of stimulating melanogenesis [87]; hence, the extraction method may play a decisive role for the finally assessed activity. In this vein, we have previously shown that *G. glabra* ethyl acetate and methanol extracts (using ASE) were able to inhibit tyrosinase activity and melanogenesis [88]. On the other hand, in the present study, the anti-tyrosinase activity was extracted with the SFE method, while the ASE extract (using water and isopropanol as solvent) proved to be inactive (Table 8). Regarding the two extracts from plants of the genus *Pistacia* (*P. lentiscus* and *P. terebinthus*), there are also reports in the literature supporting their tyrosinase inhibitory activity [77,89]. Notably, this is the first report regarding the tyrosinase inhibitory activity of a *C. creticus* ssp. *eriocephalus* extract, although there are publications regarding other subspecies of the genus *Cistus* [90,91], including *C*. *parviflorus* [92]. Similarly, the ability of *A. cephalonica* and *P. idaeum* extracts to suppress tyrosinase activity is shown for the first time, since there were reports in the literature only for other subspecies of the *Abies* [93,94] and *Polygonum* [95,96] genera. The antimelanogenic activities of *S. sediforme*, *H. emerus*, and *A. unedo* extracts are also in agreement with previous reports by us and others [16,88,97,98,99]. Essential oils from *J. oxycedrus* have been shown to possess tyrosinase inhibitory activity [43], as well as extracts from other subspecies of the *Juniperus* genus [100,101,102], though—to our knowledge—there are no such reports regarding *J. turbinata*.

Targeting cellular mechanisms responsible for proteome quality control by natural products is a promising approach to delay cellular senescence and/or *in vivo* aging [103]. Thus, we examined the activity of the two main proteolytic pathways of the cell, the Ubiquitin-Proteasome Pathway (UPP) and the Autophagy-Lysosome Pathway (ALP) [104]. Specifically, we assessed the main proteasome peptidase activity, the chymotrypsin–like activity (CT-L/β5), and the most abundant lysosomal proteases’ activity, that of cathepsins. Regarding the proteasomal activity, 12 extracts (9 ASE and 3 SFE) were tested, 75% of which were found to decrease peptidase action, while only two of them had a positive effect. Proteasome is considered a crucial target for therapeutic intervention; both its downregulation and upregulation can be beneficial for the progression delay of age-related diseases like cancer and neurodegeneration [105]. Therefore, proteasome inhibitors have gained a lot of interest in their efficacy against inflammation and cancer [106,107,108]. Extracts isolated using the ASE technique from *Origanum vulgare* and *Sedum sediforme* have been tested for their effect on proteasome activity in our previous study [16], where they were found not to change significantly or to mildly decrease the activity; these findings agree with the results of the present study. In addition, proteasome inhibition can lead to activation of the ALP pathway, indirectly mediating the maintenance of protein homeostasis [109]. Extracts obtained with the ASE technique from *Pistacia lentiscus* and *Pistacia terebinthus* were found here, for the first time, to activate the proteasome. Regarding autophagy modulation, it is known to be inhibited in some pathological conditions, contributing to the disease deterioration, while it may be permissive in others, towards pathogenesis [110]. From the extracts examined in the present study, two were found to activate cathepsins’ activity, one isolated from *Pistachia terebinthus* and one from *Hippocrepis emerus*, both obtained with the ASE technique. Suppression of cathepsins’ activity was observed after treatment with the majority of the extracts. Autophagy has been suggested to delay the skin photoaging process caused by UV irradiation through degradation of oxidized lipids and metabolic wastes; thus, natural products inducing the activity of this pathway may be promising agents for the cosmeceutical and medicinal industries [111,112]. However, some cathepsins can be secreted and remain functionally active outside of the lysosome. Specifically, extracellular cathepsins contribute to the degradation of many extracellular matrix components, like elastin, collagen, proteoglycans, and fibronectins, and participate in physiological processes, like wound healing and bone remodeling. They have been reported to be upregulated in pathological states, where uncontrolled cell proliferation, invasion, and differentiation or elastin depletion, caused by aberrant ECM dynamics, are observed [113,114,115]. Therefore, a number of natural products have been reported as selective inhibitors of some cathepsins [116,117].

Activities of all extracts assessed in the present study are summarized in a heatmap (Figure 7), which could prove valuable for the selection of specific targets for more detailed analysis. Moreover, certain extracts seem to combine multiple activities very useful for the cosmetic industry. In particular, extract 5A and extract 40A scored high in six of the bioactivity assays performed, extract 1A scored high in five of them, and extracts 8A, 21A, 22A, and 30A scored high in four assays (Figure 7).

To correlate chemical composition with biological activity, extracts found to exhibit multiple bioactivities were analyzed using LC-HRMS/MS for qualitative identification of the derived compounds. To that end, dereplication strategies were employed, i.e., spectrometric and chromatographic features, HRMS/MS fragmentation patterns, and in-house and open databases, as well as literature information. Compounds were tentatively identified based on accurate mass (Δm < 5 ppm), RDBeq., and chromatographic and spectrometric characteristics. Twelve (12) metabolites were annotated in extract 1A from *Abies cephalonica*, including dicarboxylic acids, flavonoids, abeosides and abietane-type diterpenes (Supplementary Table S2A). Notably, compounds such as 15-hydroxy-7-oxodehydroabietic acid ([M+H-H_2_O]^+^, *m*/*z* = 313.1796), 15-hydroxydehydroabietic acid ([M+H-H_2_O]^+^, *m*/*z* = 299.2003), and 7-oxodehydroabietic acid ([M-H]^−^, *m*/*z* = 313.1808), have been previously reported within the *Abies* genus [118,119]. Several of these diterpenes are known to possess moderate antibacterial and anti-inflammatory properties [120]. Additionally, abeoside D ([M-H+FA]^−^, *m*/*z* = 507.1715) and abeoside G ([M-H+FA]^−^, *m*/*z* = 519.2081) have also been previously identified and isolated from the trunk of *Abies* species. Abeoside G, along with abeosides A and B, have demonstrated strong anti-neuroinflammatory activity [121].

In extract 5A (*Cistus parviflorus)*, 19 metabolites were tentatively identified, including quinic acid ([M-H]^−^, *m*/*z* = 191.0563), epigallocatechin ([M-H]^−^, *m*/*z* = 305.0670), quercetin-3-glucoside ([M-H]^−^, *m*/*z* = 463.0883), and tiliroside ([M-H]^−^, *m*/*z* = 593.1296), among others (Supplementary Table S2B). Overall, the available literature on *C. parviflorus* is significantly more limited compared to other *Cistus* species. Nevertheless, it has been reported that *C. parviflorus* exhibits stronger tyrosinase inhibition than other species [92].

As for extract 8A (*E. parviflorum*), 20 metabolites were annotated. Derivatives of phenolic acids like gallic acid ([M-H]^−^, *m*/*z* = 169.0141), gentisic acid ([M-H]^−^, *m*/*z* = 153.0194), ellatannins such as oenothein B ([M-2H]^2−^, *m*/*z* = 1567/783.0679), flavonoids (e.g., quercetin [M+H]^+^, *m*/*z* = 303.0496, kaempferol [M+H]^+^, *m*/*z* = 287.0549) and flavonoid glycosides (e.g., myricetin 3-O-rhamnoside [M-H]^−^, *m*/*z* = 463.0874, astragalin [M-H]^−^, *m*/*z* = 447.0923) were tentatively identified (Supplementary Table S2C). These compounds have previously been reported within the *Epilobium* genus and in general in the Onagraceae family [122,123]. Notably, several studies have suggested that oenothein B may play a key role in the pharmacological activities attributed to *Epilobium* species [124].

Twelve metabolites were annotated in extract 21A (*Pistacia terebinthus*), including hydrolysable tannins (3,5-Di-O-galloylquinic acid, [M-H]^−^, *m*/*z* = 495.0779), flavonoids (Myricetin-O-glucuronide, [M-H]^−^, *m*/*z* = 493.0618 and Myricitrin, [M-H]^−^, *m*/*z* = 463.0877), and fatty acids ((9Z)-5,8,11-trihydroxyoctadec-9-enoic acid, [M-H]^−^, *m*/*z* = 327.2178) (Supplementary Table S2D). These compounds have been previously identified in this species [89]. Traditionally, several parts of the tree have been used in folk medicine. Previous studies have shown that methanolic extracts of *P*. *terebinthus* exhibit strong antioxidant activity and high metal-chelating capacity. Moreover, the extracts inhibited key metabolic and neurological enzymes (AChE, BChE, α-amylase, α-glucosidase, tyrosinase), highlighting their potential for neuroprotective, metabolic, and cosmetic applications [125].

In extract 22A (*Sedum sediforme*), 17 metabolites were annotated, most of which belong to the class of flavonoid glycosides (Supplementary Table S2E). Notably, compounds identified include gentisic acid 5-O-glucoside ([M-H]^−^, *m*/*z* = 315.0722), quercitrin ([M-H]^−^, *m*/*z* = 447.0930), eriodictyol-4′-O-glucoside ([M-H]^−^, *m*/*z* = 449.1088), and tricin 7-glucoside ([M-H]^−^, *m*/*z* = 491.1196), all of which have previously been reported in methanolic extracts of *S. sediforme* [126]. Furthermore, earlier studies have shown that methanolic extracts from the aerial parts of *S. sediforme* exhibit strong inhibitory effects on 5-lipoxygenase, suggesting an anti-inflammatory potential, along with notable antioxidant activity [127,128].

Regarding *C. creticus* ssp. *eriocephalus* extract 30A, 23 metabolites were annotated (Supplementary Table S2F). The majority belong to the flavonoid class, including tiliroside ([M-H]^−^, *m*/*z* = 593.1299), acacetin ([M-H]^−^, *m*/*z* = 283.0615), luteolin ([M-H]^−^, *m*/*z* = 285.0406), and kaempferol ([M-H]^−^, *m*/*z* = 285.0406). Additionally, phenolic acids such as protocatechuic acid ([M-H]^−^, *m*/*z* = 153.0195) and hydroxybenzoate esters like urlanneoside ([M-H]^−^, *m*/*z* = 285.0619) were detected. These metabolites are commonly found within the *Cistus* genus [129]. However, similar to *C. parviflorus*, the literature on *C. creticus* ssp. *eriocephalus* remains limited, with most existing studies focusing primarily on its essential oil composition. In traditional medicine, various *Cistus* species have been widely used for their anti-inflammatory, hemostatic, tonic, anti-diabetic, and antispasmodic properties [130,131].

Literature on *Polygonum idaeum* remains limited, which complicates compound identification. Nevertheless, 21 compounds were annotated in the extract 40A (Supplementary Table S2G), primarily belonging to the class of benzoic acid derivatives (e.g., gentisic acid [M-H]^−^, *m*/*z* = 153.0194, 4-hydroxybenzoic acid [M-H]^−^, *m*/*z* = 137.0245), polyphenols (e.g., gallic acid [M-H]^−^, *m*/*z* = 169.0143 and rosmarinic acid ([M-H]^−^, *m*/*z* = 359.0771), flavonoid glycosides (e.g., avicularin [M-H]^−^, *m*/*z* = 433.0778), and fatty acids (e.g., stearidonic acid [M+H]^+^, *m*/*z* = 277.2157). These metabolites have been previously reported in other *Polygonum* species [132]. Interestingly, other species of the genus have demonstrated photoprotective effects. For instance, *P. multiflorum* extracellular vesicle-like nanovesicles have been explored as a novel natural therapeutic agent against photoaging due to the presence of several antioxidant compounds [133].

## 5. Conclusions

In the present study, 104 extracts from 41 genera and 49 different species of plants endemic in Greece were evaluated regarding a broad spectrum of biological activities related to skin aging. Many of them were found to possess interesting properties in terms of antioxidant potential, photoprotective capacity, antimelanogenic activity, and inhibition of enzymes such as collagenase, elastase, and proteasomal enzymes. In general, extracts obtained through ASE exhibited higher activities than those of extracts obtained through SFE. Seven extracts showed multiple activities, being highly effective in at least four different assays. Their chemical composition and literature data supported our present findings. Due to the broad spectrum of assays used in the current study, a selection strategy had to be employed in some of the assays, a fact that can be considered as a limitation of the study. Moreover, the multifarious nature of the extracts may lead to masking of certain activities, e.g., during the *in vivo* zebrafish embryo melanogenesis assay, the presence of a toxic compound in the whole extract could mask the identification of a compound of interest due to using the maximum tolerated concentration. Future studies will be designed employing a bioactivity-driven subfractionation in order to enrich the extracts in the compound of interest or even isolate the single bioactive compound.

In conclusion, novel properties of various plants of the Greek flora identified in the present study, along with the existing knowledge, will prove valuable for their potential use in interventions against skin aging and in cosmetic applications.

## Figures and Tables

**Table 1:** Origin of the plant material.

N°	Plant	Family	Part of the Plant Collected	Codes
1	*Abies cephalonica*	Pinaceae	Stems and bark	COSM_1
2	*Achillea millefolium*	Asteraceae	Inflorescences	COSM_2
3	*Arbutus unedo*	Ericaceae	Branches and leaves	COSM_3
4	*Ceratonia siliqua*	Fabaceae	Aerial parts	COSM_4
5	*Cistus parviflorus*	Cistaceae	Aerial parts in full flowering	COSM_5
6	*Cistus salviifolius*	Cistaceae	Aerial parts in flowering	COSM_6
7	*Epilobium dodonaei*	Onagraceae	Aerial parts	COSM_7
8	*Epilobium parviflorum*	Onagraceae	Aerial parts	COSM_8
9	*Eryngium amorginum*	Apiaceae	Aerial parts	COSM_9
10	*Glycyrrhiza glabra*	Fabaceae	Roots	COSM_10
11	*Hypericum perforatum*	Guttiferae	Aerial parts	COSM_11
12	*Inula candida* ssp. *candida*	Asteraceae	Aerial parts	COSM_12
13	*Malva sylvestris*	Malvaceae	Aerial parts	COSM_13
14	*Melissa officinalis*	Lamiaceae	Aerial parts	COSM_14
15	*Mentha pulegium*	Lamiaceae	Aerial parts	COSM_15
16	*Nepeta spruneri*	Lamiaceae	Whole plant	COSM_16
17	*Origanum dictamnus*	Lamiaceae	Flowering stems	COSM_17
18	*Origanum majorana*	Lamiaceae	Aerial parts in flowering	COSM_18
19	*Origanum vulgare* ssp. *hirtum*	Lamiaceae	Aerial parts	COSM_19
20	*Pistacia lentiscus*	Anacardiaceae	Branches and leaves	COSM_20
21	*Pistacia terebinthus*	Anacardiaceae	Branches and leaves	COSM_21
22	*Sedum sediforme*	Crassulaceae	Aerial parts	COSM_22
23	*Acantholimon graecum*	Plumbaginaceae	Aerial parts in full flowering	COSM_23
24	*Anchusa azurea*	Boraginaceae	Whole plant in full flowering	COSM_24
25	*Armeria canescens*	Plumbaginaceae	Whole plant	COSM_25
26	*Artemisia absinthium*	Asteraceae	Aerial parts	COSM_26
27	*Atractylis gummifera Β*	Asteraceae	Taproots	COSM_27
28	*Atractylis gummifera A*	Asteraceae	Aerial parts	COSM_28
29	*Cercis siliquastrum*	Fabaceae	Aerial parts and some fruits	COSM_29
30	*Cistus creticus* ssp. *eriocephalus*	Cistaceae	Aerial parts	COSM_30
31	*Caridothymus capitatus*	Lamiaceae	Aerial parts in full flowering	COSM_31
32	*Cotinus coggygria*	Anacardiaceae	Stems and leaves	COSM_32
33	*Crithmum maritimum*	Apiaceae	Stems and leaves before flowering	COSM_33
34	*Dorycnium hirsutum*	Fabaceae	Aerial parts, flowers, and few fruits	COSM_34
35	*Genista halacsyi*	Fabaceae	Aerial parts with fruits	COSM_35
36	*Helianthemum salicifolium*	Cistaceae	Aerial parts	COSM_36
37	*Hippocrepis emerus* ssp. *emeroides*	Fabaceae	Aerial parts at the end of flowering	COSM_37
38	*Onosma erecta* ssp. *erecta*	Boraginaceae	Whole plant in full flowering	COSM_38
39	*Paeonia mascula* ssp. *hellenica*	Paeoniaceae	Stems and leaves (without seeds)	COSM_39
40	*Polygonum idaeum*	Polygonaceae	Whole plant and roots	COSM_40
41	*Salvia officinalis*	Lamiaceae	Aerial parts at the end of flowering	COSM_41
42	*Salvia pomifera*	Lamiaceae	Aerial parts in flowering	COSM_42
43	*Salvia sclarea*	Lamiaceae	Aerial parts in flowering	COSM_43
44	*Sambucus nigra*	Adoxaceae	Stems, leaves and flowers	COSM_44
45	*Silybum marianum*	Asteraceae	Aerial parts at the beginning of flowering	COSM_45
46	*Opuntia ficus indiga*	Cactaceae	Liophilized fruits	COSM_46
47	*Citrus medica*	Rutaceae	Liophilized fruit	COSM_47
48	*Ebenus cretica*	Fabaceae	Aerial parts in flowering	COSM_48
49	*Juniperus oxycedrus* ssp. *deltoides*	Cupressaceae	Branches and leaves	COSM_49
50	*Juniperus oxycedrus* ssp. *deltoides*	Cupressaceae	Fruits	COSM_50
51	*Juniperus turbinata*	Cupressaceae	Branches and leaves	COSM_51
52	*Juniperus turbinata*	Cupressaceae	Fruits	COSM_52

**Table 2:** Highest non-cytotoxic extract concentration for human skin fibroblasts (DSF) and keratinocytes (HaCaT).

Extract Code	Concentration (μg/mL)		Extract Code	Concentration (μg/mL)		Extract Code	Concentration (μg/mL)	
	DSF	HaCaT		DSF	HaCaT		DSF	HaCaT
1A	4	20	18B	20	100	36A	100	100
1B	100	100	19A	100	100	36B	20	100
2A	4	100	19B	100	20	37A	100	100
2B	20	100	20A	100	20	37B	100	100
3A	20	20	20B	100	4	38A	100	100
3B	20	100	21A	20	20	38B	20	100
4A	20	20	21B	100	20	39A	4	20
4Β	20	20	22A	20	20	39B	100	100
5A	100	4	22B	4	100	40A	100	100
5Β	20	100	23A	100	20	40B	100	100
6A	0.8	100	23B	4	100	41A	20	100
6B	20	100	24A	100	100	41B	20	100
7A	100	20	24B	100	100	42A	20	100
7B	20	100	25A	100	20	42B	100	100
8A	20	100	25B	20	100	43A	20	100
8B	100	100	26A	100	100	43B	20	20
9A	100	100	26B	100	100	44A	0.16	100
9B	20	20	27A	100	100	44B	100	100
10A	100	100	27B	20	20	45A	20	100
10B	20	20	28A	100	100	45B	100	100
11A	100	100	28B	100	100	46A	4	100
11B	20	20	29A	100	100	46B	0.032	0.16
12A	100	100	29B	20	100	47A	4	4
12B	20	20	30A	100	20	47B	100	4
13A	100	20	30B	100	100	48A	100	100
13B	100	100	31A	100	100	48B	100	100
14A	100	100	31B	100	100	49A	100	100
14B	100	100	32A	100	100	49B	0.16	0.032
15A	100	20	32B	100	4	50A	4	4
15B	100	20	33A	100	100	50B	0.16	0.032
16A	100	100	33B	100	100	51A	100	100
16B	20	100	34A	100	100	51B	100	0.032
17A	100	100	34B	100	100	52A	100	100
17B	100	100	35A	100	100	52B	20	4
18A	100	100	35B	20	100			

**Table 3:** Scavenging of DPPH by the test extracts.

Extract Code	Inhibition (% of Control)		Extract Code	Inhibition (% of Control)		Extract Code	Inhibition (% of Control)	
	Mean	SD		Mean	SD		Mean	SD
1A	27.0	1.5	19A	61.1	1.8	37A	105.9	5.3
1B	105.4	0.9	19B	80.9	0.6	37B	98.1	3.9
2A	62.8	0.9	20A	51.7	2.1	38A	71.9	3.2
2B	102.6	3.3	20B	102.8	1.2	38B	88.4	5.7
3A	45.2	3.9	21A	47.5	0.6	39A	76.8	1.9
3B	109.8	1.2	21B	75.2	5.3	39B	99.0	2.8
4A	31.9	3.3	22A	26.3	0.6	40A	15.1	0.6
4Β	105.4	0.8	22B	64.4	1.1	40B	93.6	1.7
5A	26.3	1.2	23A	24.1	1.1	41A	28.7	0.7
5Β	97.9	3.3	23B	97.3	1.4	41B	86.6	4.1
6A	24.4	0.6	24A	73.1	3.1	42A	25.2	0.5
6B	108.7	0.3	24B	100.5	2.2	42B	72.6	3.3
7A	22.0	1.5	25A	32.8	0.6	43A	36.4	2.2
7B	108.3	0.3	25B	101.3	1.5	43B	105.4	1.7
8A	27.9	2.7	26A	94.8	0.9	44A	88.0	5.1
8B	103.2	3.6	26B	105.3	1.2	44B	97.6	0.3
9A	68.5	2.1	27A	101.1	1.6	45A	92.9	1.6
9B	114.5	0.9	27B	107.6	0.3	45B	100.9	5.6
10A	85.1	0.3	28A	102.3	0.5	46A	93.2	10.5
10B	79.3	1.2	28B	106.2	0.9	46B	95.8	0.9
11A	32.9	0.3	29A	39.7	0.1	47A	99.9	4.8
11B	108.7	0.3	29B	95.7	1.0	47B	95.1	3.7
12A	55.0	0.3	30A	13.2	0.8	48A	75.8	2.6
12B	96.2	1.2	30B	103.7	1.4	48B	98.7	4.5
13A	107.1	3.9	31A	64.7	0.9	49A	34.6	1.7
13B	89.9	1.5	31B	94.0	1.6	49B	106.8	1.3
14A	73.6	1.8	32A	40.0	0.1	50A	106.4	2.6
14B	107.5	1.8	32B	101.8	1.6	50B	98.4	3.1
15A	63.8	1.2	33A	98.6	2.4	51A	39.2	1.3
15B	85.1	1.8	33B	99.1	0.9	51B	92.4	8.7
16A	87.7	2.1	34A	70.1	1.5	52A	80.2	3.8
16B	111.7	1.5	34B	99.9	3.0	52B	104.1	14.9
17A	64.0	0.3	35A	95.6	0.2	Trolox ^a^	18.3	0.6
17B	80.0	0.3	35B	98.6	2.0	Vehicle	100.0	7.9
18A	66.5	0.0	36A	51.8	5.4			
18B	101.9	0.3	36B	93.3	1.0			

^a^ 100 μM.

**Table 4:** Suppression of ROS levels by the extracts (**A**) in human skin fibroblast cultures (**B**) in human keratinocyte cultures.

(A)								
Extract Code	Inhibition (% of Control)		Extract Code	Inhibition (% of Control)		Extract Code	Inhibition (% of Control)	
	Mean	SD		Mean	SD		Mean	SD
1A	44.9	0.5	19A	69.8	2.2	37A	75.1	0.3
1B	67.5	1.1	19B	62.4	2.4	37B	87.8	2.5
2A	66.4	1.1	20A	59.9	2.2	38A	50.7	1.1
2B	66.2	0.5	20B	68.4	0.5	38B	63.4	1.4
3A	86.7	0.3	21A	70.5	1.3	39A	54.1	4.6
3B	74.9	2.5	21B	65.4	1.6	39B	85.4	4.2
4A	100.6	1.4	22A	68.6	0.4	40A	49.9	0.7
4Β	100.4	1.3	22B	82.0	1.1	40B	85.5	3.2
5A	49.5	0.9	23A	65.6	3.9	41A	65.9	2.7
5Β	95.1	1.3	23B	64.1	0.6	41B	47.2	0.3
6A	64.4	0.8	24A	95.8	1.9	42A	51.1	0.6
6B	94.8	1.1	24B	94.3	1.9	42B	55.8	0.9
7A	74.8	2.6	25A	103.2	1.8	43A	87.9	0.5
7B	75.3	4.3	25B	98.9	2.5	43B	48.4	0.9
8A	77.8	2.2	26A	94.1	2.9	44A	45.7	0.7
8B	79.4	0.2	26B	51.0	0.5	44B	77.4	2.9
9A	54.6	1.0	27A	50.1	1.6	45A	63.9	1.8
9B	98.2	2.0	27B	51.5	0.7	45B	92.3	3.3
10A	52.2	0.4	28A	49.6	0.9	46A	54.7	1.6
10B	50.4	0.7	28B	48.7	0.7	46B	55.5	2.0
11A	51.1	0.2	29A	51.8	1.1	47A	97.7	2.1
11B	100.2	3.3	29B	91.6	2.0	47B	47.8	5.1
12A	97.1	1.7	30A	54.0	1.4	48A	45.2	0.9
12B	84.0	0.4	30B	70.7	0.8	48B	46.2	0.5
13A	78.4	2.1	31A	48.3	0.9	49A	96.2	0.7
13B	78.5	1.2	31B	103.6	0.5	49B	40.3	0.8
14A	66.5	0.9	32A	109.9	4.3	50A	99.5	0.7
14B	76.0	1.0	32B	93.2	1.6	50B	89.1	0.5
15A	68.0	1.2	33A	64.2	0.4	51A	96.8	2.0
15B	85.2	1.1	33B	79.2	3.4	51B	42.6	1.1
16A	59.7	0.9	34A	51.0	0.7	52A	42.1	0.4
16B	71.7	0.7	34B	79.4	2.6	52B	83.0	2.1
17A	58.5	1.6	35A	63.8	1.5	Trolox ^a^	58.8	1.9
17B	69.5	4.6	35B	76.5	2.5	Vehicle	100.0	2.3
18A	70.1	1.4	36A	65.4	1.3			
18B	49.8	0.2	36B	56.8	1.0			
(B)								
Extract Code	Inhibition (% of Control)		Extract Code	Inhibition (% of Control)		Extract Code	Inhibition (% of Control)	
	Mean	SD		Mean	SD		Mean	SD
1A	69.2	2.7	20A	97.2	1.4	39A	71.6	3.1
1B	81.0	0.2	20B	108.1	3.5	39B	84.7	1.0
2A	92.2	1.4	21A	130.7	2.6	40A	61.3	0.6
2B	95.6	1.5	21B	101.7	2.3	40B	71.7	1.7
3A	105.0	4.8	22A	127.2	0.1	41A	71.2	1.2
3B	100.1	2.1	22B	64.9	0.7	41B	76.6	0.8
4A	110.9	2.6	23A	61.7	0.8	42A	76.0	2.1
4Β	99.8	2.6	23B	77.7	1.0	42B	71.4	1.7
5A	83.5	0.6	24A	51.8	0.2	43A	73.8	1.4
5Β	77.5	0.9	24B	79.8	1.0	43B	73.7	0.5
6A	75.0	2.0	25A	74.4	1.2	44A	73.5	0.6
6B	87.3	0.8	25B	74.1	1.7	44B	81.4	0.8
7A	106.5	2.0	26A	60.2	0.7	45A	82.9	1.7
7B	99.3	1.6	26B	72.5	2.6	45B	88.6	0.6
8A	112.6	4.4	27A	59.9	0.9	46A	88.7	2.4
8B	85.5	2.4	27B	106.1	2.6	46B	99.5	2.2
9A	91.2	1.5	28A	69.9	1.3	47A	88.2	1.6
9B	105.0	1.0	28B	89.8	2.7	47B	77.0	1.9
10A	71.2	0.2	29A	63.4	1.1	48A	54.9	0.8
10B	87.6	2.7	29B	68.2	0.8	48B	73.5	0.8
11A	70.9	2.8	30A	61.5	1.6	49A	56.5	1.5
11B	97.5	1.2	30B	74.6	1.6	49B	90.9	1.4
12A	116.8	4.6	31A	67.8	3.0	50A	65.9	1.1
12B	69.6	1.1	31B	59.8	1.2	50B	112.2	3.3
13A	89.5	0.5	32A	72.0	2.0	51A	61.3	1.7
13B	102.8	1.4	32B	107.9	1.5	51B	85.6	2.5
14A	99.3	1.3	33A	64.8	2.3	52A	62.9	0.6
14B	99.8	0.7	33B	85.0	2.9	52B	93.8	7.9
15A	107.1	0.6	34A	65.9	1.3	NAC ^a^	50.3	1.7
15B	92.5	0.4	34B	81.6	1.5	Vehicle	100.0	1.5
16A	95.6	0.4	35A	74.3	0.3			
16B	102.3	2.9	35B	78.4	1.0			
17A	97.1	2.0	36A	64.8	0.9			
17B	95.7	1.3	36B	90.4	0.5			
18A	89.7	1.3	37A	90.8	1.2			
18B	89.3	1.0	37B	94.1	0.8			
19A	105.2	1.5	38A	57.7	0.5			
19B	87.6	0.8	38B	76.1	1.0			

^a^ 100 μM; N-acetyl-cysteine (5 mM).

**Table 5:** Percent inhibition of UVB-induced cytotoxicity.

Extract Code	% Inhibition		Extract Code	% Inhibition		Extract Code	% Inhibition	
	DSF	HaCaT		DSF	HaCaT		DSF	HaCaT
1A	41	14	18B	7	18	36A	5	4
1B	7	0	19A	12	6	36B	4	0
2A	10	0	19B	2	3	37A	5	11
2B	0	0	20A	33	7	37B	0	7
3A	62	8	20B	0	0	38A	6	9
3B	16	7	21A	43	8	38B	0	7
4A	17	8	21B	0	0	39A	4	9
4Β	6	10	22A	27	30	39B	0	3
5A	64	65	22B	39	6	40A	86	24
5Β	5	18	23A	48	29	40B	19	0
6A	28	32	23B	0	0	41A	21	2
6B	0	5	24A	0	4	41B	11	3
7A	14	36	24B	5	1	42A	15	7
7B	0	16	25A	19	9	42B	13	6
8A	88	22	25B	19	0	43A	18	8
8B	7	21	26A	29	0	43B	10	1
9A	2	14	26B	0	0	44A	14	4
9B	0	13	27A	7	0	44B	8	12
10A	0	7	27B	7	2	45A	13	3
10B	2	1	28A	16	13	45B	8	3
11A	56	12	28B	3	8	46A	7	5
11B	4	6	29A	26	14	46B	6	0
12A	0	7	29B	33	9	47A	5	4
12B	14	0	30A	54	15	47B	0	5
13A	9	14	30B	0	7	48A	13	7
13B	9	19	31A	6	5	48B	0	4
14A	8	7	31B	0	7	49A	28	0
14B	0	12	32A	7	0	49B	6	0
15A	33	14	32B	0	5	50A	7	0
15B	2	8	33A	27	5	50B	0	1
16A	17	17	33B	6	1	51A	13	0
16B	7	17	34A	11	6	51B	0	0
17A	38	38	34B	0	3	52A	9	0
17B	13	38	35A	23	0	52B	6	0
18A	18	18	35B	0	1			

**Table 6:** Inhibition of collagenase activity by active ^a^ extracts.

Extract Code	Inhibition (% of Control)	
	Mean	SD
1A	23.3	2.1
2A	9.7	2.7
3A	35.1	2.2
4A	18.9	9.7
5A	31.2	3.1
7A	45.8	3.7
8A	26.7	2.6
10A	3.3	6.7
20A	42.1	3.3
21A	40.2	2.0
30A	1.2	1.7
39A	22.1	3.7
40A	21.0	10.8
47A	18.3	9.9
49A	22.3	2.1
50A	19.1	4.8
51A	27.0	1.3
EGCG ^b^	92.2	0.1

^a^ Extracts not shown were devoid of collagenase activity; ^b^ epigallocatechin gallate 1 mM.

**Table 7:** Inhibition of elastase activity by active ^a^ extracts.

Extract Code	Inhibition (% of Control)	
	Mean	SD
5A	25.5	5.7
15A	2.0	0.2
15B	6.0	1.0
16A	9.0	1.0
17A	1.0	0.1
17B	4.0	1.0
18A	4.0	0.4
18B	6.0	0.2
19A	17.0	1.0
19B	9.0	1.0
20A	60.0	0.3
20B	7.0	0.1
21A	45.0	0.1
21B	7.0	0.3
22A	24.0	1.0
22B	1.0	0.1
23A	4.0	1.0
23B	3.0	0.3
24A	4.0	1.0
25A	2.0	0.4
25B	3.0	1.0
26A	3.0	0.4
26B	6.0	1.3
28A	21.0	11.4
40A	24.0	0.1
46A	2.0	0.2
50A	7.0	2.0
MeO-Suc-AAPV-CMK ^b^	97.0	3.0

^a^ Extracts not shown were devoid of elastase activity; ^b^ N-(Methoxysuccinyl)-Ala-Ala-Pro-Val-chloromethyl ketone (750 μM).

**Table 8:** Inhibition of tyrosinase activity by the extracts.

Extract Code	Inhibition (% of Control)		Extract Code	Inhibition (% of Control)		Extract Code	Inhibition (% of Control)	
	Mean	SD		Mean	SD		Mean	SD
1A	62.1	1.3	20A	74.9	0.1	39A	^a^	
1B	^a^		20B	16.4	0.2	39B	^a^	
2A	7.2	0.1	21A	65.2	1.5	40A	52.7	1.5
2B	^a^		21B	51.7	1	40B	^a^	
3A	41.4	2.9	22A	65.3	0.8	41A	20.5	0.3
3B	2.7	0.04	22B	^a^		41B	30.8	1.2
4A	28.8	0.2	23A	34.2	0.2	42A	32.7	0.2
4Β	^a^		23B	8.1	0.2	42B	16.4	0.3
5A	56.1	2.2	24A	22.8	0.2	43A	9.8	0.7
5Β	4.7	0.03	24B	^a^		43B	^a^	
6A	7.1	0.2	25A	41.1	0.4	44A	14.6	0.6
6B	^a^		25B	7.5	0.4	44B	^a^	
7A	^a^		26A	12.2	0.1	45A	^a^	
7B	^a^		26B	^a^		45B	^a^	
8A	^a^		27A	^a^		46A	^a^	
8B	^a^		27B	^a^		46B	^a^	
9A	^a^		28A	3	0.03	47A	5.1	0.04
9B	^a^		28B	^a^		47B	^a^	
10A	^a^		29A	22.3	2.2	48A	20.8	0.02
10B	99.1	0.2	29B	2.5	0.1	48B	23.8	0.4
11A	3.6	0.16	30A	69.3	5.8	49A	47.6	0.1
11B	^a^		30B	^a^		49B	23.5	0.3
12A	6.2	0.24	31A	13.1	0.16	50A	^a^	
12B	^a^		31B	^a^		50B	8.3	0.2
13A	2.7	0.2	32A	0.2	0.01	51A	49.3	0.1
13B	^a^		32B	^a^		51B	31.8	1.9
14A	20	0.2	33A	^a^		52A	11.6	0.7
14B	^a^		33B	^a^		52B	5.4	0.1
15A	16.8	0.1	34A	30.7	0.7	Kojic acid ^b^	89.8	1.4
15B	^a^		34B	^a^				
16A	6.4	0.1	35A	10.7	0.1			
16B	^a^		35B	^a^				
17A	18.1	0.2	36A	^a^				
17B	1.9	0.1	36B	5.6	0.2			
18A	15.8	0.7	37A	64.3	0.5			
18B	33.6	0.6	37B	60.7	0.6			
19A	15.9	0.8	38A	5.5	0.2			
19B	^a^		38B	^a^				

^a^ Devoid of tyrosinase activity; ^b^ 100 μM.

## Data Availability

Data are contained within the article or Supplementary Materials.

## Supplementary Materials

The following supporting information can be downloaded at: https://www.mdpi.com/article/10.3390/antiox14070824/s1, Table S1: Sequences of the primers used, Table S2: Dereplication tables.
