# Supplementary material for: Screening of a Plant Extract Library from the Greek Flora for Biological Activities Related to Anti-Aging Applications
Source: Antioxidants (Basel). 2025 Jul 4;14(7):824. doi: 10.3390/antiox14070824 (PMC12291661; doi:10.3390/antiox14070824)
Supplement: Supplementary file 1 [file antioxidants-14-00824-s001.zip › antioxidants-3694123-supplementary.pdf]

Article

Screening of a plant extract library from the Greek flora for biological activities related to anti-aging applications

Harris Pratsinis[1], Despoina D. Gianniou[2], Gabriela Belen Lemus Ringele[3], Adamantia Agalou[4], Asimina Fotopoulou[1], Xanthippi P. Louka[2], Christos Nastos[5], Aikaterini Argyropoulou[3], Dimitris Michailidis[5], Antonia Theodoridi[4], Ioanna Eleftheriadou[4], Adamantia Papadopoulou[1], Sentiljana Gumeni[2], Stavros Beteinakis[3], Konstantina Karamanou[1], Eleni Mavrogonatou[1], Georgios Stavropoulos[7], Dimitris Beis[4,6], Maria Halabalaki[3], Ioannis P. Trougakos[2] and Dimitris Kletsas[1,*]

1 Laboratory of Cell Proliferation & Ageing, Institute of Biosciences & Applications, NCSR "Demokritos", Athens 15341, Greece; hprats@bio.demokritos.gr
2 Department of Cell Biology and Biophysics, Faculty of Biology, National and Kapodistrian University of Athens, 15784 Athens, Greece; itrougakos@biol.uoa.gr
3 Division of Pharmacognosy and Natural Products Chemistry, Department of Pharmacy, National and Kapodistrian University of Athens, 15771 Athens, Greece; mariahal@pharm.uoa.gr
4 Center for Clinical, Experimental Surgery & Translational Research, Biomedical Research Foundation, Academy of Athens, 11527 Athens, Greece; dbeis@bioacademy.gr
5 PharmaGnose S.A., 57th km Athens-Lamia Athens-Lamia Road, 32011 Oinofyta Viotia, Greece; michailidis@pharmagnose.com
6 Laboratory of Biological Chemistry, Faculty of Medicine, School of Health Sciences, University of Ioannina, 45110 Ioannina, Greece; dbeis@uoi.gr
7 Korres S.A.—Natural Products, 57th km Athens-Lamia Athens-Lamia Road, 32011 Oinofyta Viotia, Greece; giorgos.stavropoulos@korres.com

* Correspondence: dkletsas@bio.demokritos.gr; Tel.: +30 210 6503583

Supplementary information

 https://doi.org/10.3390/antiox14070824

Supplementary Table S1: Sequences of the primers used

Target Gene	NCBI Reference Sequence	Direction	Sequence	Length (bp)	Annealing T (°C)	Fragment Size (bp)
NFE2L2	NM_006164.5	Forward	5′-CATCCAGTCAGAAACCAGTGG -3′	21	60	85
		Reverse	5′-GCAGTCATCAAAGTACAAAGCAT-3′	23		
GAPDH	NM_002046.7	Forward	5′-CCACATCGCTCAGACACCAT-3′	20	60	179
		Reverse	5′-CCATGGGTGGAATCATATTGGAAC-3′	25		
NQO1	NM_000903.3	Forward	5′-AGCAGACGCCCGAATTCAAA-3′	20	60	95
		Reverse	5′-AGAGGCTGCTTGGAGCAAAA-3′	20		
TXNRD1	NM_001093771.3	Forward	5′-TTGGAGTGCGCTGGATTTCT-3′	20	60	99
		Reverse	5′-TTTGTTGGCCATGTCCTGGT-3′	20		

Dereplication tables

Supplementary Table S2A: Annotated compounds of extract 1A (*Abies cephalonica*) using HRMS in negative and positive ionization mode

Peak ID	Retention time (min)	Molecular formula	Exp. *m/z* detected [M-H]$^-$	Exp. *m/z* detected [M+H]$^+$	Delta (ppm)	RDBeq.	Annotated Compound
1	0.69	$C_7H_{11}O_6$	191.0561	-	-0.18	2.5	Quinic acid
2	1.51	$C_{15}H_{13}O_7$	305.0667	-	0.11	9.5	Epigallocatechin
3	2.79	$C_{15}H_{13}O_7$	305.0668	-	0.51	9.5	Gallocatechin
4	3.15	$C_{15}H_{13}O_6$	289.072	-	0.79	9.5	Epicatechin
5	3.49	$C_{21}H_{31}O_{14}$	507.1715 [M-H+FA]$^-$	-	-0.76	6.5	Abeoside D
6	3.97	$C_{19}H_{25}O_{12}$	445.135	-	-0.31	7.5	Abeoside A or B
7	4.55	$C_8H_{13}O_4$	173.0819	-	-0.39	2.5	Suberic acid
8	5.4	$C_9H_{15}O_4$	187.0974	-	-1.23	2.5	Azelaic acid
9	5.81	$C_{23}H_{35}O_{13}$	519.2081 [M-H+FA]$^-$	-	-0.47	6.5	Abeoside G
10	7.73	$C_{20}H_{25}O_3$	-	313.1796 [M+H-H_2O]+	-0.6216	8.5	15-hydroxy-7-oxodehydroabietic acid
11	8.82	$C_{20}H_{28}O_2$	-	299.2005 [M+H-H_2O]+	-0.1876	7.5	15-Hydroxydehydroabietic acid
12	10.32	$C_{20}H_{27}O_3$	-	315.1952	-0.794	7.5	7-Oxoabieta-8,11,13-trien-18-oic acid

Supplementary Table S2B: Annotated compounds of extract 5A (*Cistus parviflorus*) using HRMS in negative and positive ionization mode

Peak ID	Retention time (min)	Molecular formula	Exp. *m/z* detected [M-H]-	Exp. *m/z* detected [M+H]+	Delta (ppm)	RDBeq.	Annotated Compound
1	0.68	$C_7H_{11}O_6$	191.0563	-	0.93	2.5	Quinic acid
2	1.06	$C_7H_5O_5$	169.0143	-	0.57	5.5	Gallic acid
3	1.33	$C_{13}H_{15}O_9$	315.0724	-	0.83	6.5	Gentisic acid 5-O-glucoside
4	1.50	$C_{15}H_{13}O_7$	305.0670	-	0.91	9.5	Epigallocatechin
5	2.47	$C_{21}H_{23}O_{11}$	451.1251	-	1.12	10.5	Catechin glycoside
6	3.15	$C_{15}H_{13}O_6$	289.0721	-	1.11	9.5	Catechin
7	3.66	$C_{21}H_{23}O_{10}$	435.1296	-	-0.21	10.5	Phlorizin
8	3.84	$C_{22}H_{17}O_{11}$	457.0779	-	0.67	14.5	Epigallocatechin gallate
9	4.6	$C_{21}H_{31}O_{12}$	475.1821	-	-0.002	6.5	Cistanoside E
10	4.64	$C_{21}H_{19}O_{12}$	463.0883	-	0.25	12.5	Quercetin 3-glucoside
11	4.71	$C_{15}H_{11}O_6$	-	287.0551	0.34	10.5	Kaempferol
12	4.71	$C_{27}H_{29}O_{15}$	593.1509	-	-0.53	13.5	C-hexosyl-C-pentosyl methyl flavonoid
13	5.03	$C_{21}H_{19}O_{11}$	447.0932	-	-0.25	12.5	Kaempferol 3-galactoside
14	6.08	$C_{30}H_{25}O_{13}$	593.1296	595.1442	-0.81	18.5	Tiliroside
15	6.23	$C_{30}H_{25}O_{13}$	593.1298	-	-0.40	18.5	Tiliroside isomer 1
16	7.02	$C_{18}H_{31}O_5$	327.2178	-	0.32	3.5	(10E,15Z)-9,12,13-Trihydroxyoctadeca-10,15-dienoic acid
17	7.4	$C_{18}H_{33}O_5$	329.2333	-	-0.03	2.5	(9Z)-5,8,11-Trihydroxyoctadec-9-enoic acid
18	7.92	$C_{18}H_{15}O_8$	359.0775	-	0.8481	11.5	4',5,7-Trihydroxy 3,6,8-trimethoxyflavone
19	9.68	$C_{19}H_{19}O_7$	-	359.1126	0.22	10.5	Quercetin-3,7,3',4'-tetramethyl ether

Supplementary Table S2C: Annotated compounds of extract 8A (*Epilobium parviflorum*) using HRMS in negative and positive ionization mode

Peak ID	Retention time (min)	Molecular formula	Exp. *m/z* detected $[M-H]^-$	Exp. *m/z* detected $[M+H]^+$		Delta (ppm)	RDBeq.	Annotated Compound
1	0.68	$C_{13}H_{15}O_{10}$	331.0672	-	0.30	6.5	1	6-O-Galloyl-glucose
2	1.07	$C_7H_5O_5$	169.0141	-	-0.69	5.5	2	Gallic acid
3	1.79	$C_7H_5O_4$	153.0194	-	0.60	5.5	3	Gentisic acid
4	2.21	$C_{13}H_{11}O_9$	311.0412	-	1.00	8.5	4	Caftaric acid
5	2.82	$C_{34}H_{23}O_{22}$	1567/783.0679 $[M-2H]^{2-}$	-	-0.98	23.5	5	Oenothein B
6	3.60	$C_{14}H_{13}O_9$	325.0564 $[M-H-C_7H_6O_5]^-$	-	-0.33	8.5	6	3,4-Di-O-galloylquinic acid
7	4.21	$C_{21}H_{19}O_{13}$	479.0829	-	-0.41	12.5	7	Myricetin 3-galactopyranoside
8	4.46	$C_{14}H_5O_8$	300.9988	-	-0.59	12.5	8	Ellagic acid
9	4.58	$C_{21}H_{19}O_{12}$	463.0874	-	-1.72	12.5	9	Myricetin-3-O-rhamnoside
10	4.58	$C_{15}H_{11}O_8$	-	319.0442	-2.14	10.5	10	Myricetin
11	4.91	$C_{21}H_{19}O_{11}$	447.0929	-	-0.80	12.5	11	Quercitrin
12	5.07	$C_{21}H_{19}O_{11}$	447.0923	-	-0.46	12.5	12	Astragalin
13	5.07	$C_{15}H_{11}O_7$	-	303.0496	-1.21	10.5	13	Quercetin
14	5.4	$C_9H_{15}O_4$	187.0975	-	-0.25	2.5	14	Azelaic acid
15	5.48	$C_{21}H_{19}O_{10}$	431.0981	-	-0.52	12.5	15	Kaempferol-3-O-rhamnoside
16	5.48	$C_{15}H_{11}O_6$	-	287.0549	-0.28	10.5	16	Kaempferol
17	7.01	$C_{18}H_{31}O_5$	327.2174	-	-0.79	3.5	17	Trihydroxy octadecadienoic acid isomer
18	7.41	$C_{18}H_{33}O_5$	329.2333	-	-0.21	2.5	18	Trihydroxyoctadecenoic acid isomer
19	7.59	$C_{16}H_{31}O_4$	287.2228	-	-0.01	1.5	19	Dihydroxyhexadecanoic acid
20	10.14	$C_{16}H_{29}O_4$	285.2073	-	0.60	2.5	20	Hexadecanoic acid

 https://doi.org/10.3390/antiox14070824

Supplementary Table S2D: Annotated compounds of extract 21A (*Pistacia terebinthus*) using HRMS in negative and positive ionization mode

Peak ID	Retention time (min)	Molecular formula	Exp. *m/z* detected $[M\text{-}H]^-$	Exp. *m/z* detected $[M+H]^+$		Delta (ppm)	RDBeq.	Annotated Compound
1	0.69	$C_7H_{11}O_6$	191.0559	-	-1.30	2.5	1	Quinic acid
2	1.04	$C_{14}H_{15}O_{10}$	343.0665 [M-H-$C_7H_4O_4$]-	-	-1.57	7.5	2	3,4-Di-O-galloylquinic acid
3	2.61	$C_{21}H_{19}O_{14}$	495.0779	-	-0.29	12.5	3	3,5-Di-O-galloylquinic acid
4	3.13	$C_8H_7O_5$	183.0297	-	-1.27	5.5	4	Dihydroxy-methoxybenzoic acid
5	4.11	$C_{21}H_{17}O_{14}$	493.0618	-	-1.17	13.5	5	Myricetin-O-glucuronide
6	4.22	$C_{21}H_{17}O_{14}$	493.0615	-	-1.17	13.5	6	Myricetin-O-glucuronide isomer
7	4.57	$C_{21}H_{19}O_{12}$	463.0877	465.1026	-1.12	12.5	7	Myricitrin
8	5.09	$C_{21}H_{19}O_{11}$	447.0931	-	-0.32	12.5	8	n-Propyl gallate
9	5.33	$C_{10}H_{11}O_5$	211.061	-	-0.76	5.5	9	Oleuropein
10	5.62	$C_{25}H_{31}O_{13}$	539.1767	-	-0.58	10.5	10	(10E,15Z)-9,12,13-Trihydroxyoctadeca-10,15-dienoic acid
11	7	$C_{18}H_{31}O_5$	327.2178	-	0.23	3.5	11	(9Z)-5,8,11-Trihydroxyoctadec-9-enoic acid
12	7.4	$C_{18}H_{33}O_5$	329.2332	-	-0.58	2.5	12	n-Propyl gallate

 https://doi.org/10.3390/antiox14070824

Supplementary Table S2E: Annotated compounds of extract 22A (*Sedum Sediforme*) using HRMS in negative and positive ionization mode

**Peak ID**	**Retention time (min)**	**Molecular formula**	**Exp. *m/z* detected** [M-H]$^-$	**Exp. *m/z* detected** [M+H]$^+$		**Delta (ppm)**	**RDBeq.**	**Annotated Compound**
1	0.69	$C_7H_5O_5$	169.0143	-	0.21	5.5	1	Gallic acid
2	1.32	$C_{13}H_{15}O_9$	315.0722	-	0.05	6.5	2	Gentisic acid 5-O-glucoside
3	1.77	$C_7H_5O_4$	153.0194	-	0.51	5.5	3	Gentisic acid
4	1.92	$C_{14}H_{19}O_{10}$	347.0984	-	-0.03	5.5	4	Picraquassioside D
5	3.4	$C_{15}H_{19}O_{10}$	359.0981	-	-0.79	6.5	5	1-O-(3-Hydroxy-4,5-dimethoxybenzoyl)hexopyranose
6	3.44	$C_8H_7O_3$	151.0401	-	0.01	5.5	6	3-Methoxybenzoic acid
7	3.89	$C_{27}H_{29}O_{15}$	593.1508	-	-0.63	13.5	7	5,7-Dihydroxy-3-(4-hydroxyphenyl)-6,8-bis[3,4,5-trihydroxy-6-(hydroxymethyl)oxan-2-yl]chromen-4-one
8	4.57	$C_{21}H_{19}O_{12}$	463.0873	465.1026	-1.85	12.5	8	Myricitrin
9	5.06	$C_{21}H_{19}O_{11}$	447.093	449.1078	-0.59	12.5	9	Quercitrin
10	5.16	$C_{21}H_{21}O_{11}$	449.1088	451.1238	-0.31	11.5	10	Eriodictyol-4'-O-glucoside
11	5.4	$C_9H_{15}O_4$	187.0975	-	-0.58	2.5	11	Azelaic acid
12	5.56	$C_{17}H_{23}O_9$	371.1345	-	-0.71	6.5	12	Syringin
13	5.98	$C_{15}H_{11}O_6$	287.0555	289.0700	-2.30	10.5	13	3,4,2',4',6'-Pentahydroxychalcone
14	6.08	$C_{22}H_{21}O_{12}$	477.1033	-	-1.21	12.5	14	2-(3,4-Dihydroxyphenyl)-5-hydroxy-7-methoxy-4-oxo-4H-chromen-3-yl hexopyranoside
15	6.46	$C_{23}H_{23}O_{12}$	491.1196	-	0.20	12.5	15	Tricin 7-glucoside
16	6.64	$C_{15}H_{11}O_5$	271.0613	-	0.41	10.5	16	Naringenin
17	7.62	$C_{16}H_{11}O_7$	315.051	-	-0.02	11.5	17	Rhamnetin

 https://doi.org/10.3390/antiox14070824

Supplementary Table S2F: Annotated compounds of extract 30A (*Cistus creticus* ssp. *eriocephalus*) using HRMS in negative and positive ionization mode

Peak ID	Retention time (min)	Molecular formula	Exp. *m/z* detected [M-H]$^-$	Exp. *m/z* detected [M+H]$^+$		Delta (ppm)	RDBeq.	Annotated Compound
1	1.14	$C_7H_{11}O_6$	191.0561	-	2.5	-0.18	1	Quinic acid
2	1.6	$C_6H_7O_7$	191.0199	-	3.5	0.98	2	Isocitric acid
3	2.91	$C_{13}H_{15}O_9$	169.0145	-	6.5	0.93	3	Dihydroxybenzoic acid-glucoside
4	3.76	$C_7H_5O_4$	153.0195	-	5.5	0.80	4	Protocatechuic acid
5	4.56	$C_{12}H_{13}O_8$	285.0619	-	6.5	1.05	5	Urlanneoside
6	5.18	$C_{15}H_{13}O_6$	289.0717	-	9.5	-0.05	6	Catechin
7	6.25	$C_{21}H_{19}O_{13}$	479.0831	-	12.5	-0.09	7	Myricetin 3-O-hexoside
8	6.65	$C_{27}H_{29}O_{16}$	609.1459	-	13.5	-0.38	8	Rutin
9	6.74	$C_{14}H_5O_8$	300.999	-	12.5	0.11	9	Ellagic acid
10	6.82	$C_{21}H_{19}O_{12}$	463.0876	-	12.5	-1.39	10	Myricitrin
11	7.26	$C_{20}H_{17}O_{11}$	433.0776	-	12.5	-0.07	11	Quercetin 3-O-pentoside
12	7.4	$C_{21}H_{19}O_{11}$	447.0932	-	12.5	-0.12	12	Kaempferol 3-O-glucoside
13	8.12	$C_{15}H_{11}O_6$	287.0563	-	10.5	0.67	13	Dihydrokaempferol
14	8.89	$C_{30}H_{25}O_{13}$	593.1299	-	18.5	-0.31	14	trans-Tiliroside
15	9.06	$C_{15}H_9O_6$	285.0406	-	11.5	0.66	15	Luteolin
16	9.11	$C_{15}H_9O_7$	301.0354	-	11.5	-0.02	16	Quercetin
17	9.98	$C_{15}H_9O_5$	269.0456	-	11.5	0.29	17	Apigenin
18	10.05	$C_{15}H_{11}O_5$	271.0614	-	10.5	0.64	18	Naringenin
19	10.16	$C_{15}H_9O_6$	285.0406	-	11.5	0.66	19	Kaempferol
20	12.05	$C_{18}H_{15}O_7$	343.0824	-	11.5	0.12	20	Quercetin trimethyl ether
21	12.38	$C_{16}H_{11}O_5$	283.0615	-	11.5	0.94	21	Acacetin
22	15.11	$C_{17}H_{13}O_5$	-	299.0913	10.5	-0.41	22	Apigenin dimethyl ether
23	15.51	$C_{22}H_{33}O_3$	-	347.2581	5.5	0.13	23	Acetoxy labdane diterpenoid isomer

Supplementary Table S2G: Annotated compounds of extract 40A (*Polygonum idaeum*) using HRMS in negative and positive ionization mode

Peak ID	Retention time (min)	Molecular formula	Exp. *m/z* detected $[M-H]^-$	Exp. *m/z* detected $[M+H]^+$		Delta (ppm)	RDBeq.	Annotated Compound
1	0.9	$C_{12}H_{22}O_{11}$	377.0855 $[M+Cl]^-$	-	-0.18	1.5	1	Sucrose
2	1.45	$C_7H_5O_5$	169.0143	-	0.39	5.5	2	Gallic acid
3	2.49	$C_7H_5O_4$	153.0194	-	0.30	5.5	3	Gentisic acid
4	2.93	$C_7H_5O_3$	137.0245	-	0.64	5.5	4	4-hydroxybenzoic acid
5	3.85	$C_{14}H_{17}O_9$	329.0876	-	-0.48	6.5	5	1-O-vanilloyl-beta-D-glucose
6	4.3	$C_{15}H_{13}O_6$	289.0714	291.0862	-1.31	9.5	6	Epicatechin
7	5.59	$C_{16}H_{19}O_{10}$	371.0985	-	0.29	7.5	7	Dihydroferulic acid 4-O-glucuronide
8	6.06	$C_{20}H_{17}O_{11}$	433.0778	-	0.35	12.5	8	Avicularin
9	6.25	$C_{18}H_{15}O_8$	359.0771	-	-0.25	11.5	9	Rosmarinic acid
10	6.47	$C_9H_{15}O_4$	187.0976	-	0.15	2.5	10	Azelaic acid
11	7.37	$C_{15}H_9O_7$	301.0354	-	0.08	11.5	11	Quercetin
12	8.04	$C_{18}H_{31}O_5$	327.2177	-	-0.14	3.5	12	(10E,15Z)-9,12,13-Trihydroxyoctadeca-10,15-dienoic acid
13	8.44	$C_{18}H_{33}O_5$	329.2331	-	-0.86	2.5	13	Trihydroxy-9-octadecenoic acid isomer 1
14	9.11	$C_{18}H_{31}O_5$	327.2178	-	0.23	3.5	14	Dihydroxy-8-oxooctadec-12-enoate
15	9.25	$C_{18}H_{31}O_5$	327.2177	-	1.81	3.5	15	Trihydroxyoctadeca-12,15-dienoic acid isomer
16	11.62	$C_{16}H_{29}O_4$	285.2068	-	-0.99	2.5	16	Hexadecanedioic acid
17	12.32	$C_{18}H_{31}O_4$	311.2222	-	-1.87	3.5	17	Octadec-9-enedioic acid
18	12.32	$C_{18}H_{29}O_2$	-	277.2157	-1.83	4.5	18	Stearidonic acid
19	12.86	$C_{18}H_{33}O_3$	297.2436	-	0.24	2.5	19	Ricinoleic acid
20	13.05	$C_{18}H_{33}O_4$	313.2380	-	-1.26	2.5	20	Octadecanedioic acid
21	14.47	$C_{20}H_{37}O_4$	341.2693	-	-1.39	2.5	21	3,3,14,14-tetramethyl-hexadecanedioic acid isomer

 https://doi.org/10.3390/antiox14070824
